# Immune dysregulation and stem-like CD8^+^ T cell enrichment in type 1 diabetes pancreatic lymph nodes

**DOI:** 10.1172/JCI196445

**Published:** 2026-09-15

**Authors:** Leeana D. Peters, Howard R. Seay, Justin A. Smith, Amanda L. Posgai, Reed L. Berkowitz, Clive H. Wasserfall, Mark A. Atkinson, Rhonda Bacher, Maigan A. Brusko, Todd M. Brusko

**Affiliations:** 1 Department of Pathology, Immunology, and Laboratory Medicine, College of Medicine,; 2 Diabetes Institute,; 3 Department of Pediatrics, College of Medicine,; 4 Department of Biostatistics, College of Public Health and Health Professions, and; 5 Department of Biochemistry and Molecular Biology, College of Medicine, University of Florida, Gainesville, Florida, USA.

**Keywords:** Autoimmunity, Immunology, Diabetes, T cells, Transcriptomics

## Abstract

Effector CD8^+^ T cells are key drivers of type 1 diabetes (T1D) pathogenesis, yet questions remain regarding the molecular defects leading to altered cytotoxicity, peripheral tissue phenotype, and receptor specificity. We analyzed human pancreatic lymph nodes (pLNs) using mass cytometry and single-cell RNA-seq (scRNA-seq) with combined T cell receptor (TCR) profiling. Cytometric analysis revealed enrichment of T stem cell memory–like (TSCM-like) cells (CD8^+^CD45RA^+^CD27^+^CD28^+^CCR7^+^CXCR3^+^) in T1D pLNs. scRNA-seq indicated an elevated inflammatory cytokine gene signature (*IFITM3*, *LTB*) along with regulators of terminal differentiation (*BCL6*, *BCL3*), coupled with downregulation of exhaustion-associated genes (*DUSP2*, *NR4A2*, *TSC22D3*) in CD8^+^ T cells in T1D pLNs. Immune response enrichment analysis (IREA) indicated IL-15 signaling as a significant driver of these phenotypes. Integrated TCR and transcriptomics analysis revealed a cluster of diverse naive-like CD8^+^ T cell clones in T1D pLNs. Comparison of pLNs and pancreatic tissue slice isolates indicated sharing of effector CD8^+^ T cells, with enhanced terminal effector signatures within the pancreas relative to paired pLNs. Multiplex imaging revealed differential localization of T cell factor 1 (TCF1)- and thymocyte selection-associated high mobility group box protein (TOX)-expressing T cells in the pancreas, with islet-proximal TCF1^+^TOX^+^ cells displaying a mixture of activation and exhaustion-associated phenotypes. Thus, we provide multimodal cellular profiles enriched in T1D tissues for consideration in therapeutic targeting.

## Abstract 2

CD8^+ ^T cells are important contributors to autoimmunity in T1D, but little is known about their phenotype in disease relevant tissue. We found increased CXCR3^+^ naive-like CD8^+^ T cells in T1D pancreatic lymph node (pLN) . Using single cell sequencing and spatial profiling of human organ donor tissues, we show that CD8^+^ T cells express reduced genes related to terminal phenotypes in T1D pLN and that they acquire enhanced effector phenotype in the pancreas.

## Introduction

Genetic fine-mapping studies identified that type 1 diabetes–associated (T1D-associated) risk variants preferentially localize to enhancer regions within T and B cells (1). However, due to inaccessibility of the pancreas from living individuals, most immune cell functional studies are derived from peripheral blood, which may not accurately reflect phenotypes within the T1D target organ. Identifying the molecular basis for T1D-associated immune dysregulation within the pancreas and pancreatic draining lymph nodes (pLNs) is important for developing targeted therapies.

The role of pLNs in promoting T1D progression is incompletely characterized, but murine and human studies have implicated the pLN as an important priming site for immune activation (2–4). In the nonobese diabetic (NOD) mouse model of T1D, pLN self-renewing autoreactive T cells were found to be an important source of islet antigen–specific cytotoxic lymphocytes (5). Human studies report skewed T cell subset distributions within T1D pLN, with reduced T follicular regulatory cell frequency reported in one study (6) and increased Th17 cells in another (7). Although there are conflicting reports on circulating Treg numbers and functional deficits in patients with T1D (8, 9), multiple studies have demonstrated reduced suppressive capacity (7) and migration toward the pancreas (10) in Tregs derived from T1D pLNs.

Though peripheral blood signatures do not always correlate with those from tissues (11), there have been many important knowledge gains linking peripheral immunological signatures to clinical outcomes. Interestingly, signatures of immunotherapeutic response have converged around CD8^+^ T cells. For example, an exhausted-like CD8^+^ T cell population expressing negative regulatory molecules (including T cell immunoreceptor with Ig and ITIM domains [TIGIT] and programmed cell death 1 [PD-1]) and exhaustion markers (Killer cell lectin-like receptor G1 [KLRG1] and CD57) was associated with C-peptide preservation after alefacept (LFA3-Ig) treatment (12). Similarly, expansion of TIGIT^+^KLRG1^+^CD8^+^ T cells was found in responders to teplizumab (anti-CD3) (13). Moreover, studies in mice (5) and humans (14, 15) have implicated CD4^+^ and CD8^+^ T stem cell memory–like (TSCMs) as a potentially important reservoir for promoting pathogenic antigen–specific responses in T1D. Although clinical studies have shown clonal relationships between exhausted-like cell populations and an activated intermediate memory CD8^+^ T cell population (12), there is a paucity of information in T1D regarding the distribution of these subsets in secondary lymphatics and the pancreas, their receptor profiles, or the pathways driving these phenotypes.

Our laboratory has previously generated sorted T cell receptor (TCR) profiles from donor-matched pLN, splenic, and peripheral blood samples, revealing cell-type–dependent repertoire sharing across tissues, with CD8^+^ T cells demonstrating the greatest degree of sharing (16). Single-cell profiling enables the examination of sharing between pLN and pancreatic T cell populations with greater resolution. To understand the phenotypic and transcriptomic profile of immune cell populations in disease-relevant tissues, we performed single-cell multi-omics analysis of pLN and pancreatic T cells from donors with and without T1D. In this study, we show that circulating phenotypic signatures previously identified to correlate with T1D status or immunotherapeutic response are shared across tissue immune phenotypes. Moreover, we offer evidence that human CD8^+^ T cells possess fewer differentiated phenotypes in T1D pLNs and that effector cell populations differentiate further upon trafficking to the pancreas.

## Results

### Naive CD8^+^CXCR3^+^ T cells are enriched in T1D pLNs.

**Figure 1:**
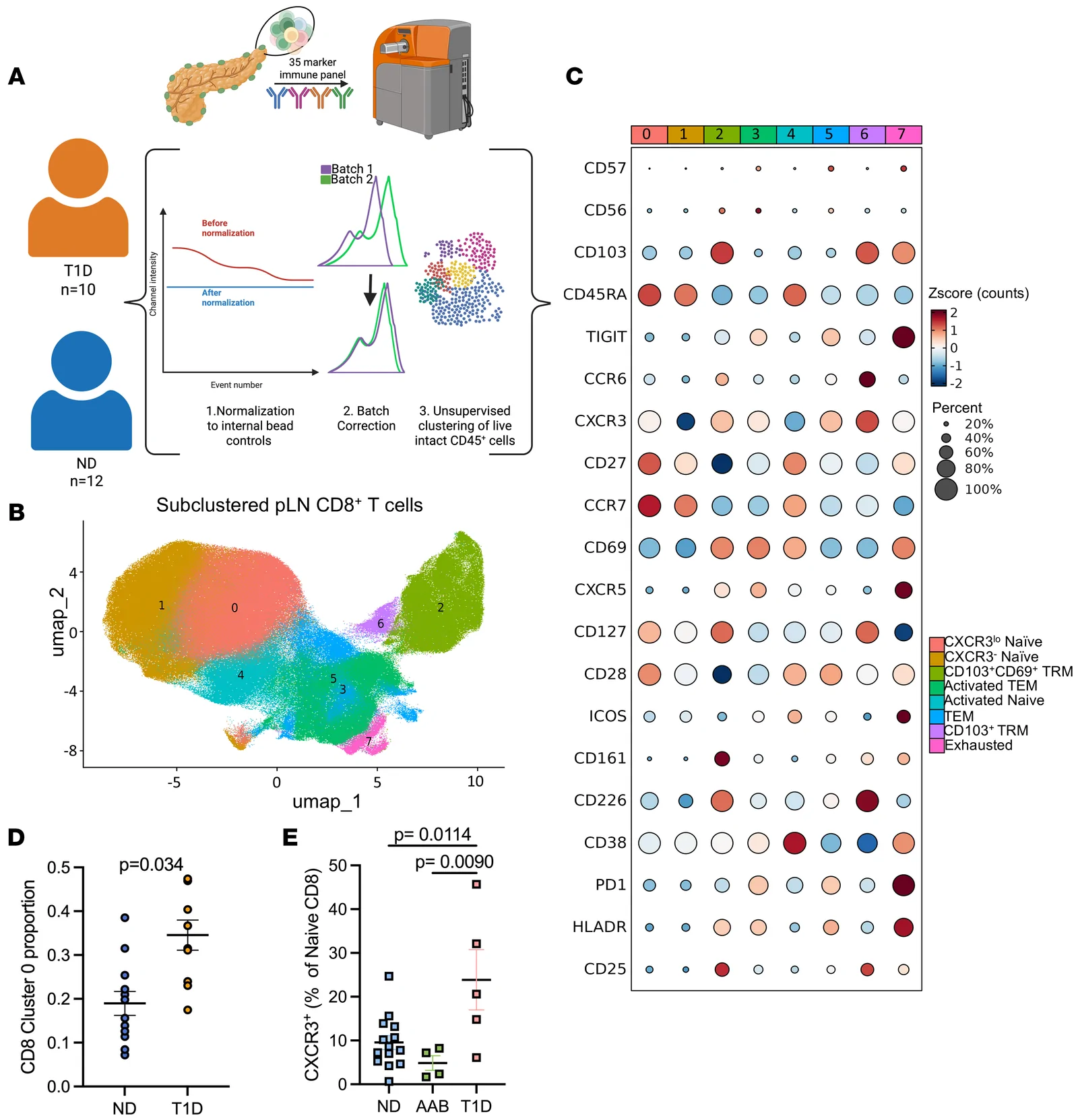
Differential abundance of CXCR3^lo^ naive CD8^+^ T cells in T1D pLNs. (**A**) Experimental workflow involving processing of pLN, staining for lineage, phenotypic, and QC markers, and data collection on a mass cytometer. After data acquisition, normalization of channels to internal bead controls used the premessa (99) package, and any confounding effects of batch or donor age on cluster composition were accounted for through incorporation as covariates in a linear model. (**B**) Uniform manifold approximation and projection (UMAP) of subclustered CD8^+^ T cells. (**C**) *Z*-scored dot plot of marker expression for each CD8^+^ T cell subcluster. (**D**) Significantly increased proportion of subcluster 0 (CXCR3^lo^ naive-like) in T1D pLN as determined by linear modeling (*n* = 22). Data are presented as the mean ± SEM. (**E**) Significantly increased percentage of naive CD8^+^ T cells expressing CXCR3 in an independent cohort of pLN donors by flow cytometry (*n* = 23). Data are presented as the mean ± SEM. The *P* value for **E** is the result of ordinary 1-way ANOVA with Tukey’s multiple test correction.

High-parameter technologies, such as cytometry time of flight (CyTOF) (12, 17, 18), are necessary to deep-phenotype immune subsets. We applied CyTOF to pLNs from 10 organ donors with T1D and 12 organ donors without diabetes (ND donors) acquired through the Network for Pancreatic Organ donors with Diabetes (nPOD) program (Supplemental Table 1 and Figure 1A; supplemental material available online with this article; https://doi.org/10.1172/JCI196445DS1). Clustering CD45^+^ immune cells revealed 11 unique cell populations spanning B cells, CD4^+^ T cells, CD8^+^ T cells, myeloid cells, and NK cells (Supplemental Figure 1A). We observed no significant differences in cluster abundance globally by clinical status (Supplemental Figure 1B).

Subclustering provided finer resolution of immune cell subsets. As 1 batch demonstrated slight, nonsignificant differences in age (Supplemental Figure 2), we compared cluster proportions using a linear model with age and batch incorporated as covariates. Subsetting and reclustering of B cells and CD4^+^ T cells yielded no significant changes in cluster abundance or marker expression by clinical status (Supplemental Figure 3). However, CD8^+^ T cell subclustering yielded 8 proteomic clusters (Figure 1B) with a T1D-associated increase in the proportion of CD8^+^ T cell cluster 0, expressing the naive T cell markers CD45RA and CCR7, the costimulatory molecules CD27 and CD28, as well as low levels of the chemokine receptor CXCR3, which confers migratory capacity toward CXCL10 (19) (Figure 1, C and D). Although we observed more variability due to the small number of donors, this finding was confirmed in an independent flow cytometry cohort consisting of 23 nPOD pLN cell isolates from ND donors, autoantibody-positive (AAb^+^) donors, and T1D donors (Supplemental Table 2) (Figure 1E and Supplemental Figure 4): T1D donors had significantly increased proportions of CXCR3^+^ cells within the naive (CD45RA^+^CCR7^+^) CD8^+^ T cell compartment compared with ND donors (*P* = 0.0114, fold change [FC] = 2.50) and AAb^+^ donors (*P* = 0.0090, FC = 4.91). Importantly, neither the frequency of this cluster nor the related flow cytometric phenotype was associated with age or disease duration (Supplemental Figure 5, A–D). These phenotypic signatures are clinically relevant in peripheral blood studies: we recently reported a T1D-associated increase in CXCR3^lo^ naive CD8^+^ T cells (20), and circulating CD8^+^ T cells with a more terminally differentiated/exhausted profile have been previously correlated with preservation of C-peptide and the response to teplizumab (12, 13).

### IL-15 drives memory maintenance and averts the exhaustion program in T1D CD8^+^ T cells.

**Figure 2:**
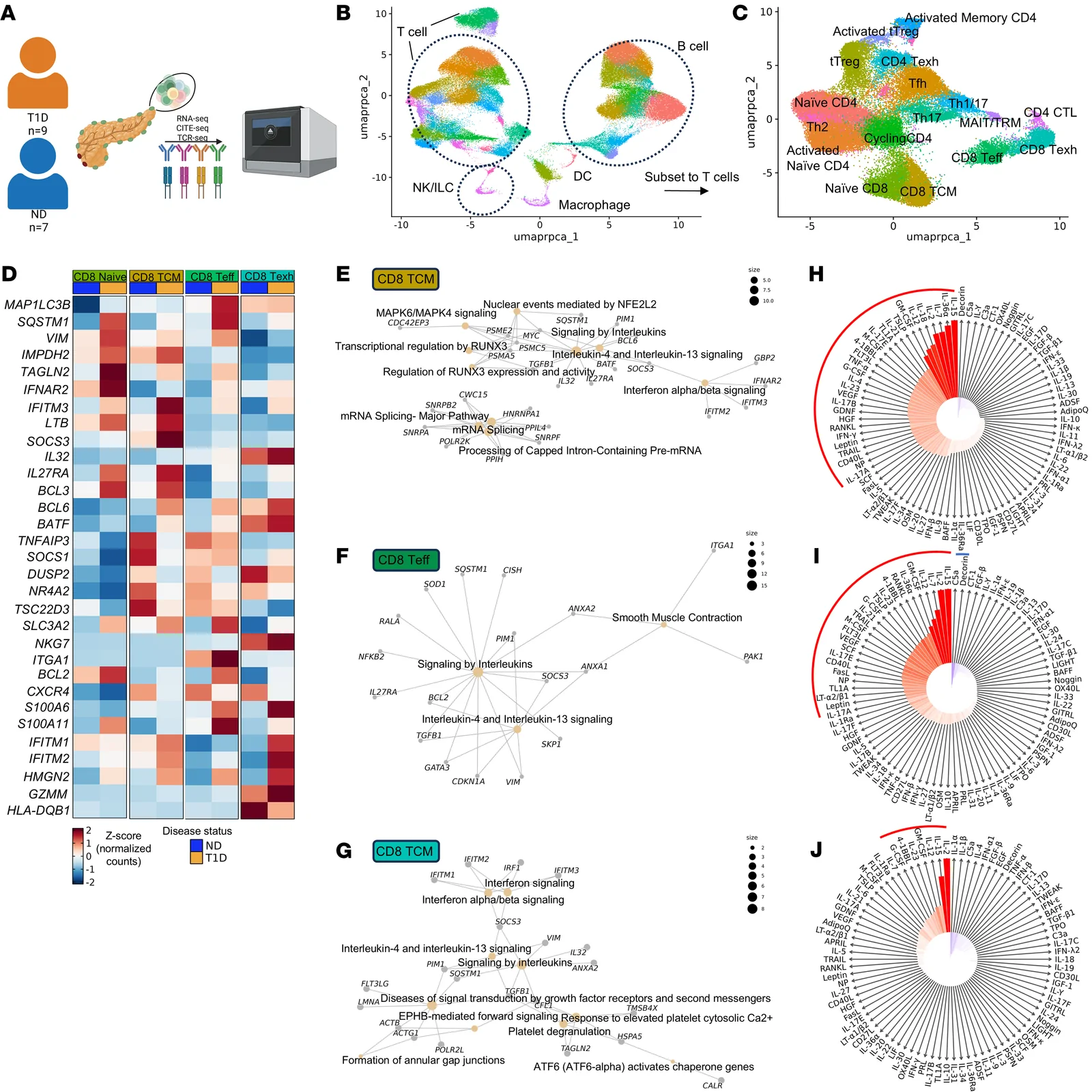
IL-15 is predicted to drive signatures of memory maintenance and effector function in T1D. (**A**) Experimental workflow for scRNA-seq of pLNs (*n* = 16). (**B**) UMAP projection of total pLN cells, which were subsetted to T cells, reintegrated with reciprocal PCA (RPCA), and reclustered, resulting in 19 T cell clusters (**C**). (**D**) Heatmap of Z-scored gene expression data showing a selection of DEGs between T1D and ND donors across naive, Tcm, Teff, and Texh CD8^+^ T cells. Network plots using DEGs in (**E**) CD8^+^ Tcm cells (**F**) CD8^+^ Teff cells, and (**G**) CD8^+^ Texh cells showing enrichment of reactome pathways in T1D. (**H**) Compass plots of IREA results using the top genes upregulated in T1D (*P* < 0.05, log_2_FC >0.26) as input for (**H**) CD8^+^ Tcm cells, (**I**) CD8^+^ Teff cells, and (**J**) CD8^+^ Texh cells. Bar height is indicative of the enrichment score; color corresponds to the FDR-adjusted *P* value (2-sided Wilcoxon rank-sum test); red or blue arcs indicate significant positive or negative enrichment, respectively. Genes highlighted on the heatmap (**D**) met log_2_FC cutoffs of 0.26 and an adjusted *P* value (MAST) cutoff of 0.05. Pathway analysis was performed with the ReactomePA package and visualized if the adjusted (hypergeometric test with Benjamini-Hochberg correction) *P* values were less than 0.05 with the Clusterprofiler package.

To explore the transcriptomic profile of the cell populations identified by both CyTOF and fluorochrome-based flow cytometry, we performed single-cell RNA-seq (scRNA-seq) with TCR-seq on donor pLNs (Figure 2A). After quality control (QC), we obtained 122,300 cells from 16 individuals (*n* = 9 T1D; *n* = 7 ND) (Supplemental Table 3) that comprised 39 clusters spanning CD4^+^ T cells, CD8^+^ T cells, B cells, NK cells, innate lymphoid cells (ILCs), monocyte/macrophages, and DCs (Figure 2B and Supplemental Figure 6, A and B). No significant differences in global cluster abundance across clinical status were detected (Supplemental Figure 6, C and D). T cell subclustering yielded 19 subclusters spanning naive, memory, and exhausted T cells, as well as Tregs (Figure 2C). Differentially expressed genes (DEGs) (FDR-corrected *P* < 0.05 and log_2_FC ≥0.26) were identified across multiple CD4^+^ T cell clusters, including T1D-associated downregulation of genes responsible for negative regulation of cytokine signaling (e.g., *TNFAIP3* [ref. 21] within naive CD4, T follicular helper [Tfh], Th1/Th17, thymic Treg [tTreg] clusters) and activation/proliferation (e.g., *JUND* [ref.22] within Tfh, tTreg, Th1/Th17, and Th17 clusters), as well as a broad increase in IFN signaling genes (e.g., *IFITM3* [ref. 23] within Th17, naive CD4, exhausted CD4, activated naive CD4, Tfh, and Th1/Th17 cells) (Supplemental Table 4).

Focusing further on pLN CD8^+^ T cell subsets, which were significantly altered in our CyTOF and flow cytometric data (Figure 1), we observed upregulation of genes involved in autophagy (*MAP1LC3B* [ref. 24], *SQSTM1* [ref. 25]), migration (*VIM*), proliferation (*IMPDH2* [ref. 26]), and cytokine production (*TAGLN2* [ref. 27]) within the naive CD8^+^ T cell cluster in T1D versus ND donors (Figure 2D). T1D CD8^+^ T central memory (Tcm) cells showed upregulation of inflammatory cytokine signaling (*IFNAR2* [ref. 28], *IFITM3*, *LTB* [ref. 29], *SOCS3* [ref. 30], *IL32* [ref. 31], *IL27RA* [ref. 32]) and regulators of terminal differentiation (*BCL3* [ref. 33], *BCL6* [ref. 34], *BATF* [ref. 35]). Both naive and Tcm CD8^+^ T cells from T1D donors displayed downregulated genes involved in the negative regulation of cytokine signaling (*TNFAIP3* [ref. 21], *SOCS1* [ref. 36]) and T cell exhaustion (*DUSP2* [ref. 37], *NR4A2* [ref. 38], *TSC22D3* [ref. 39]). In the CD8^+^ effector T (Teff) cell cluster (Figure 2D), we observed an upregulation of genes involved in effector function (*SLC3A2* [ref. 40], *NKG7* [ref. 41]), as well as migratory genes (*VIM* [ref. 42], *ITGA1* [ref. 43]) in T1D relative to ND donors. Within CD8^+^ Teff cells from T1D donors, we also noted upregulation of the antiapoptotic factor *BCL2*, which is important for the maintenance of long-lived memory cell subsets (44, 45) and downregulation of genes associated with terminal differentiation and exhaustion (*CXCR4* [ref. 46], *DUSP2* [ref. 37]). Similarly, in the exhausted CD8^+^ T cell (CD8^+^ Texh) cluster (Figure 2D), we observed upregulation of genes involved in migration (*VIM* [ref. 42]), calcium signaling/alarmins (*S100A6*, *S100A11* [ref. 47]), inflammatory cytokine response (*SOCS3*, *IFITM1*, *IFITM3*, *IFITM2* [refs. 23, 48]), and cytotoxicity (*HMGN2* [ref. 49], *GZMM*), whereas genes associated with terminal differentiation and exhaustion (*TSC22D3*, *DUSP2*, *HLA-DQB1* [ref. 50]) were downregulated in T1D donor cells. We did not detect differential expression of other canonical markers of stemness or exhaustion (i.e., *TCF7*, *LEF1*, *CD122*, *CXCR3*, *PD1*), potentially due to data sparsity or differential regulation of protein and RNA expression.

Pathway analysis using the Reactome database revealed that naive CD8^+^ T cells (Supplemental Figure 7A) in T1D samples were enriched for autophagy and mitophagy. Additionally, T1D CD8^+^ Tcm cells were enriched for IL signaling and IFN signaling (Figure 2E), while CD8^+^ Teff cells (Figure 2F) were enriched for IL signaling and smooth muscle contraction, the latter likely reflecting increased calcium signaling (*ANXA1*, *ANXA2*) (51). Last, CD8^+^ Texh cells (Figure 2G) were enriched for IFN signaling, IL signaling, and signal transduction through growth factor receptors (*TGFB1*, *FLT3LG* [ref. 52]) and second messengers (*PIM1* [ref. 53]). We then performed immune response enrichment analysis (IREA), which uses an extensive database of gene sets induced by cytokine treatment (54), to infer the cytokine milieu driving these CD8^+^ T cell phenotypes. Across the trajectory of CD8^+^ T cell differentiation, IL-15 signaling was notably enriched in T1D versus ND (Supplemental Figure 7B; Figure 2, H–J). The IL-15 pathway is known to promote stemness and restrain exhaustion-like phenotypes (55) and can synergize with other inflammatory cytokines to promote effector function (56). Collectively, these data and our prior peripheral blood findings (20) suggest Tc1 skewing as an immunological feature of T1D shared across tissues.

### CD8^+^ T cell clones possessing naive-like and TCF7^+^TOX^+^ phenotypes are more prevalent in T1D pLNs.

**Figure 3:**
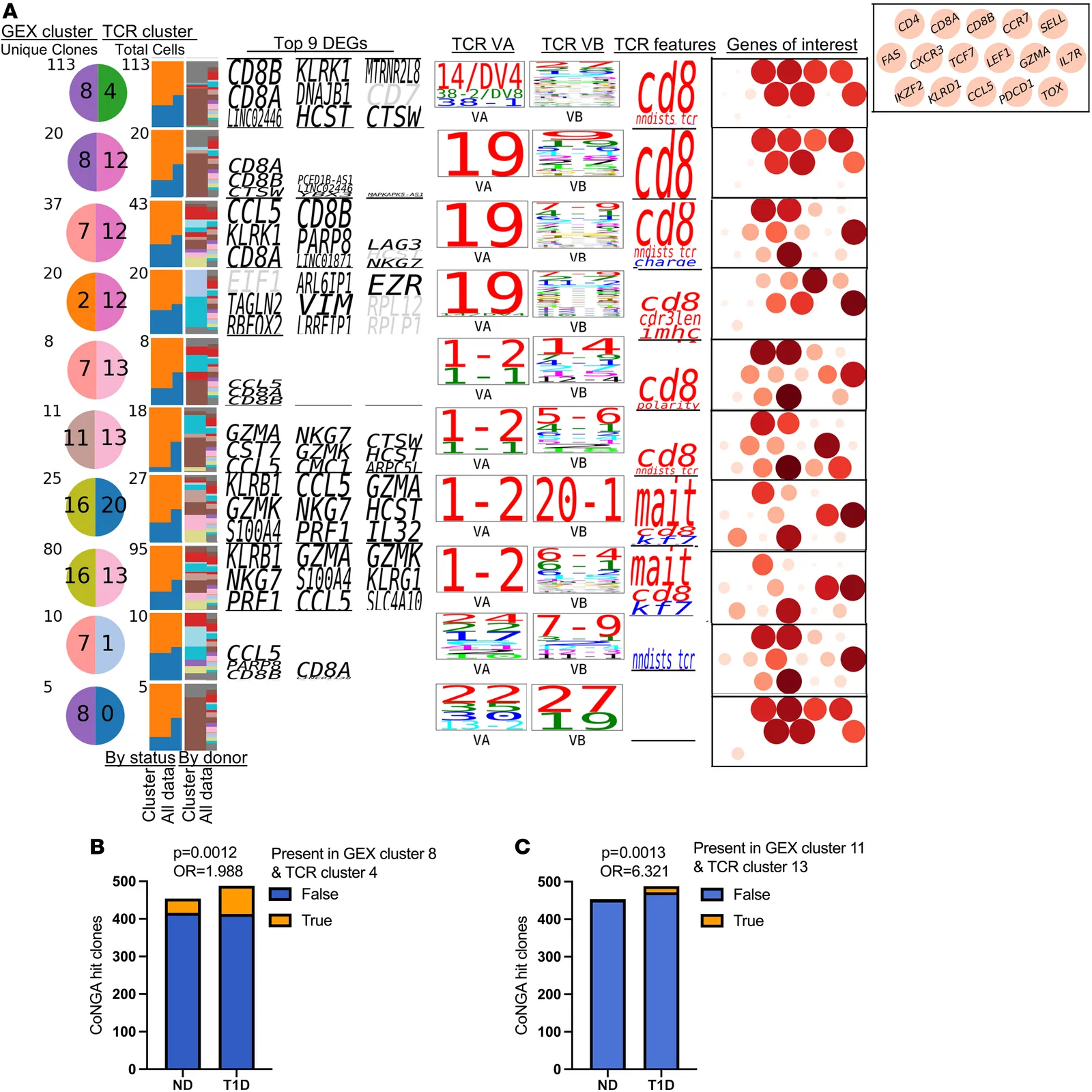
Integrative receptor and gene expression analysis highlights an enriched naive CD8^+^ T cell TCR cluster in T1D pLNs. (**A**) Gene expression and TCR graph versus graph results for CoNGA clusters with a score below 1. The left half of the semicircle indicates gene expression cluster; the right half of the semicircle indicates TCR cluster assignment. Bar plots indicate the relative proportion of T1D (orange) and ND (blue) cells for the given cluster (left) and for the entire dataset (right), followed by the same for the nPOD ID, where colors represent different donors. The top-9 DEGs are shown per cluster, as well as TCR genes and TCR sequence features (57) (TCR-seq features panel: red indicates higher and blue indicates lower scores relative to the rest of the dataset). Manually curated marker genes are shown on the far right, with red dots colored by mean expression and sized by the percentage of cells expressing the gene. DEGs were determined by Wilcoxon sum-rank test and scaled by an adjusted *P* value, where full height required a *P* value of less than 10^–6^. Fold changes of less than 2 are shown in gray. Differences in T1D-associated GEX cluster 8/TCR cluster 4 (**B**) and GEX cluster 11/TCR cluster 13 (**C**) were assessed with a 2-way Fisher’s exact test. VA, TCR-variable alpha chain; VB, TCR-variable beta chain.

Recent methods have been developed to enable joint analysis of repertoire characteristics and phenotype, namely Clonotype Neighbor Graph Analysis (CoNGA) (57), which can reveal transcripts associated with specific TCR genes and features. We applied CoNGA to our pLN dataset, which identified 35 groups with significant (CoNGA score <1) overlap in TCR and gene expression neighborhoods and a minimum of 4 cells per group (Supplemental Figure 8). In accordance with the original CoNGA publication (57), we found a stronger relationship between receptor and phenotypes in CD8^+^ T cells and invariant T cells: the majority of our CoNGA hit clusters were composed of CD8^+^ T cells, including naive, effector, and mucosal-associated invariant T (MAIT) cell clusters (Supplemental Figure 8). Among these clusters, we found 10 that were overrepresented in T1D donors (Figure 3A, left-most orange bar).

We observed that CoNGA clone hits belonging to CoNGA gene expression (GEX) cluster 8 and TCR cluster 4 were found in several T1D donors (highest frequency in new-onset donor 6550), whereas 37 of 38 clones detected in ND donors (97%) were found in a single ND donor possessing high-risk HLA alleles (HLA A*0201, HLA DRB1*0401) (Supplemental Table 5). Thus, clones belonging to these clusters were more often found in T1D donors (*P* = 0.0012, OR = 1.988) (Figure 3B). The enriched GEX cluster 8/TCR cluster 4 was composed of CD8^+^ T cells with high TCR diversity (113 unique clonotypes and 113 total cells, indicative of no clonal expansion) and high expression of *CCR7*, *SELL*, *LEF1*, *TCF7*, and *IL7R*. This naive-like cluster possessed some features suggesting prior activation, namely *KLRK1* (58), costimulatory molecule *HCST* (59) encoding DAP10, and low *CXCR3* expression. This cluster displayed a diverse TCR β chain (*TRB*) repertoire, while α chain (*TRA*) gene usage was restricted to TRAV14/DV4, TRAV38-2/DV8, and TRAV38-1 (Figure 3A). Additionally, this cluster had a low Atchley factor 4 score (blue af4 logo, TCR-seq features panel, Figure 3A), which has previously been associated with larger, more hydrophobic residues (57). GEX cluster 11/TCR cluster 13 possessed a more terminally differentiated profile, expressing cytotoxicity marker genes (*GZMA*, *GZMK*, *NKG7*), the costimulatory molecule gene *HCST*, as well as *CXCR3*, *TCF7* and *TOX* (Figure 3A). Although infrequent, this cluster was also present in multiple T1D donors, with only 2 of 18 clones being found in ND donors (Figure 3C).

We searched for T1D clones (60) in these T1D-associated CoNGA clusters based on a similarity to the known α chain complementary determining region 3 (CDR3) sequence, given recent data on potential α chain biases in autoreactive clones in T1D (61). Within the naive-like cluster, several clones shared CDR3a (allowing 1 amino acid mismatch) with known T1D clones in T1D donors, namely four GAD65-reactive clones, 2 preproinsulin-reactive (PPI-reactive) clones, and a clone that possessed a CDR3a chain shared across multiple specificities in a new-onset donor. We also found 3 clones with CDR3a matching known GAD65, ZNT8_186–194_, and PPI_16–25_ clones in a control donor with high-risk HLA alleles (Supplemental Table 5). In the more terminally differentiated cluster, we found an expanded clone (clone size of 6) matching a PPI-reactive clone, as well as sharing the α chain motif “AGGYQKVTF” present in the previously published 22.A10 (62) PPI_1-11_ clone (Supplemental Table 5). Thus, these enriched clusters may contain autoreactive specificities. Interestingly, we also noted an increase (*P* = 0.0007, OR = 2.153) in a group of expanded TCRs (GEX cluster 16, TCR cluster 13) composed of MAIT and semi-invariant T cells (*TRAV1-2* gene usage and a more diverse set of *TRB* chains) in T1D pLNs (Supplemental Figure 9).

### Shared memory T cell populations have an enhanced effector phenotype in pancreatic tissue slices.

**Figure 4:**
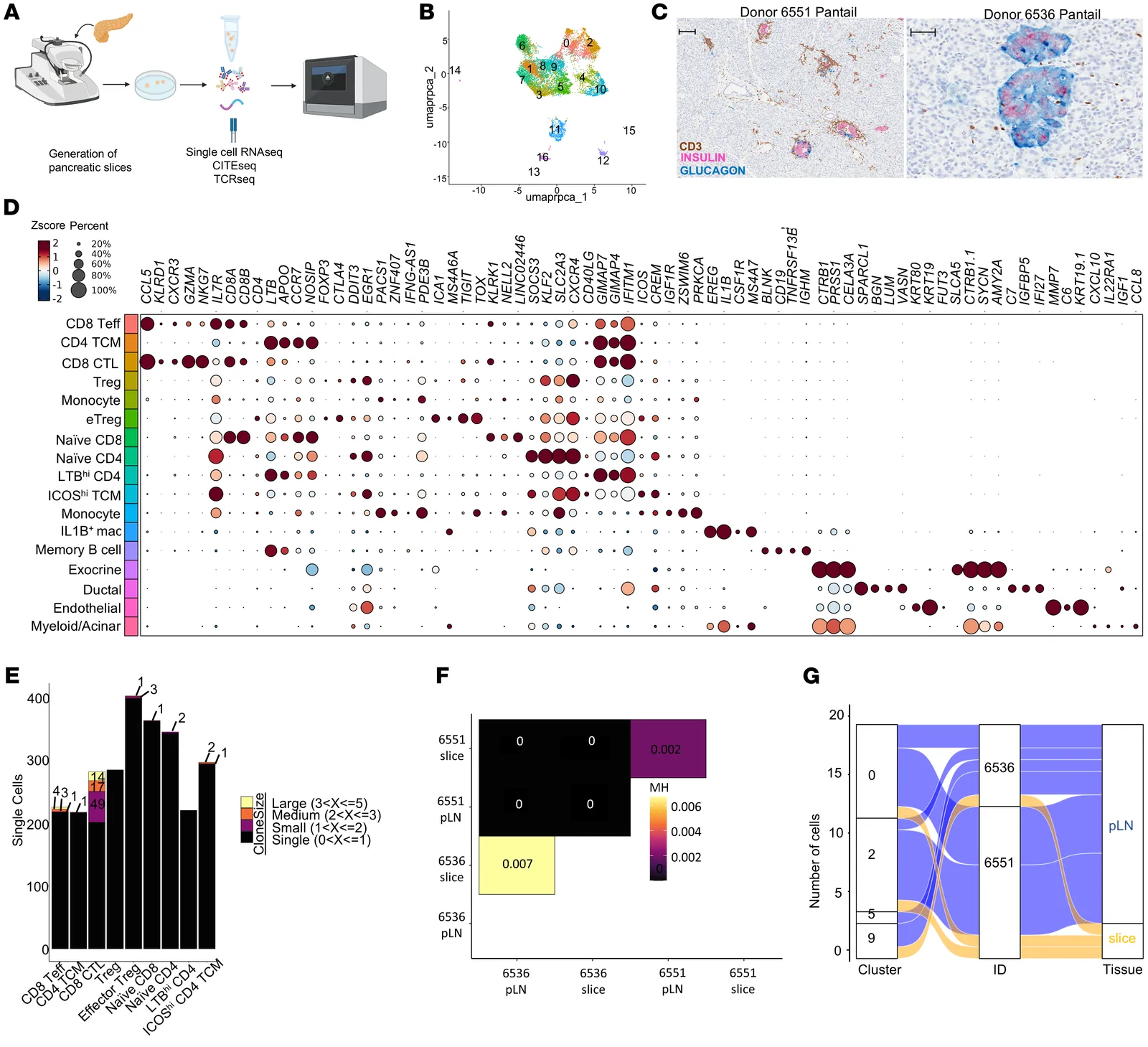
Cross-tissue clone and phenotype sharing in pLNs and pancreas. (**A**) Processing workflow of human pancreatic slices: pancreas was embedded in agarose and sliced using a vibratome to yield 150 μm slices (63, 64) and then processed to a single-cell suspension, selected for CD45^+^ cells, and run on the 10X Chromium controller. (**B**) UMAP of integrated gene expression data (*n* = 2 donors, pLN and pancreatic slices). (**C**) Histological staining (obtained from nPOD Aperio eSlide manager) for insulin (pink), glucagon (blue), and CD3 (brown) across the pancreas tail of the 2 donors with paired pancreatic slice and pLN data shows increased insulitis in donor 6551 with new-onset T1D. Scale bars: 101 mm for donor 6551 (original magnification, ×10) and 50 mm for donor 6536 (original magnification, ×20). (**D**) Dot plot showing *z*-scored gene expression per cluster in **B**, with dot size corresponding to the percentage of cells in the cluster expressing the marker. Marker genes were determined by the Wilcoxon rank-sum test. (**E**) Bar plot illustrating the distribution of expanded clones across clusters, with the majority of expanded clones localizing to memory CD8^+^ T cell cluster 0 (CD8^+^ Teff) and cluster 2 (CD8^+^ CTL). (**F**) MH metric shows limited repertoire overlap across tissues. (**G**) Alluvial plot subsetted to only shared clones showing clonal sharing between clusters 5 (eTreg) and 9 (ICOS^hi^ Tcm), and 0 (CD8^+^ Teff) and 2 (CD8^+^ CTL) across both clusters and tissues.

To investigate receptor and phenotype sharing across tissues, we performed scRNA-seq on isolated immune cells from fresh pancreatic tissue slices (63, 64) and paired pLNs (Figure 4A) from 2 donors with recent-onset T1D (nPOD 6551, duration 0.58 years; nPOD 6536, duration 4 years) with independently confirmed insulitis (65). Data integration yielded 17 clusters, including naive, memory, and effector CD4^+^ and CD8^+^ T cell populations, Tregs, B cells, and resident macrophage populations (Figure 4B). We first interrogated the TCR repertoire, and while both donors possessed insulitis, donor 6551 with newer-onset T1D possessed more extensive insulitis than did donor 6536 (Figure 4C). We found that expanded clones localized mostly to clusters 0 (CD8^+^ Teff) and 2 (CD8^+^ cytotoxic T lymphocytes [CTLs]), respectively corresponding to CD8^+^ T cell populations with lower and higher expression of cytotoxicity-related molecules (*GZMK*, *CST7*, *LTB*) (Figure 4, D and E). Clone sharing across pancreas and pLN was minimal, with Morisita-Horn (MH) values of 0.007 and 0.002 for cross-tissue overlap for donors 6536 and 6551, respectively (Figure 4F). However, even with our limited sample size, we were able to detect 6 unique clonotypes shared across tissues and clusters, with 2 clones shared across tissues (Figure 4G and Table 1). We detected an expanded CD4^+^ T cell clone in donor 6536 (cluster 9, ICOS^hi^ Tcm), as well as an expanded CD8^+^ T cell clone in donor 6551 (clusters 0 and 2 CD8^+^ Teff and CTL), which were shared between tissues. Most sharing was detected between the effector CD8 clusters 0 and 2, especially in donor 6551, as compared with donor 6536, perhaps due to reduced infiltration and increased disease duration.

**Figure 5:**
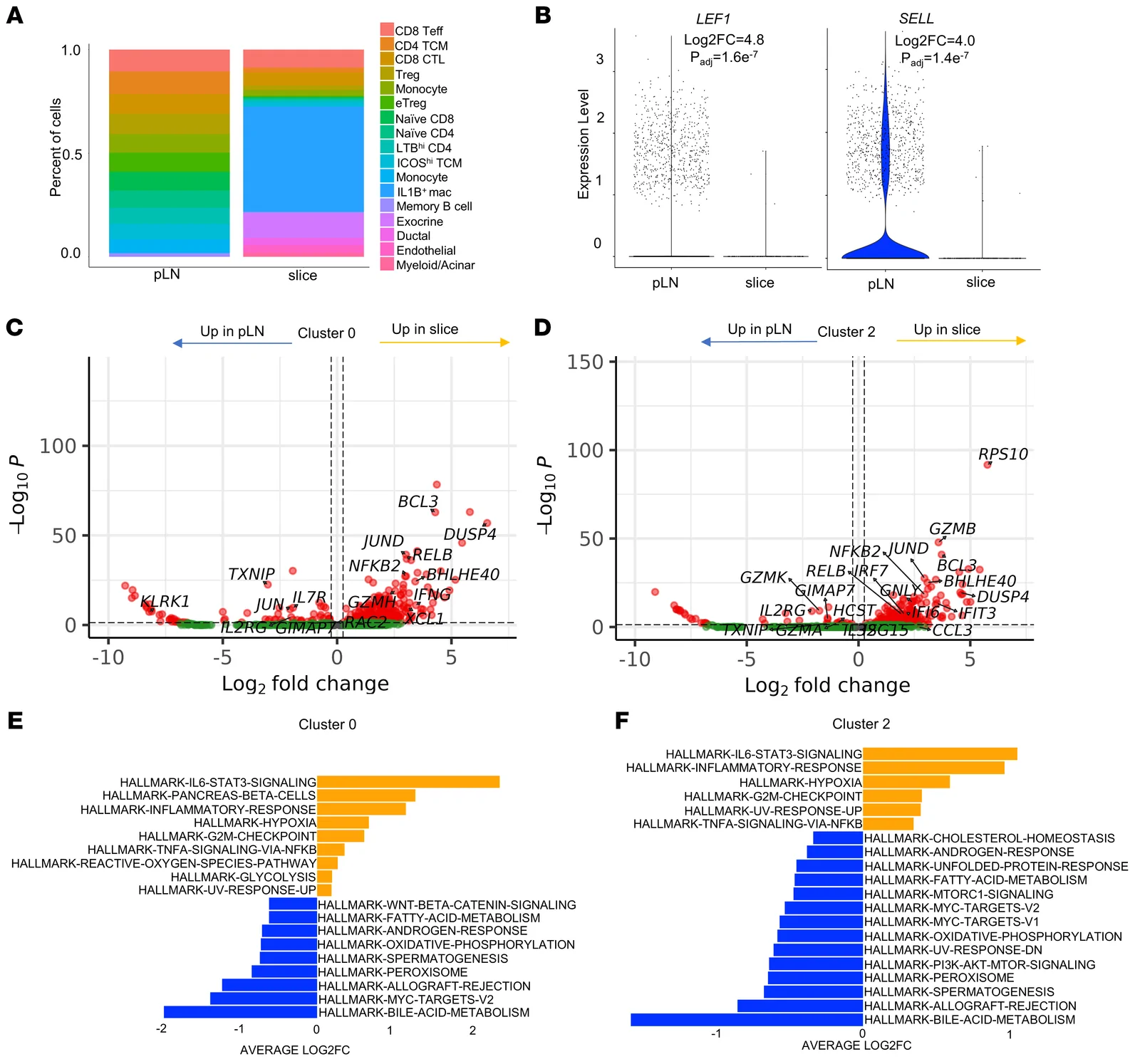
Tissue-dependent gene expression programs of memory CD8^+^ T cell populations. (**A**) Bar plot illustrating the percentage of each cluster per tissue. (**B**) Violin plots illustrating differential expression of *LEF1* and *SELL* in CD8^+^ T cells between tissues. (**C**) Volcano plot of DEGs in cluster 0 (CD8^+^ Teff) and (**D**) cluster 2 (CD8^+^ CTL) between pancreatic slices and pLNs. DEGs were defined as a log_2_FC of greater than 0.26 and a corrected *P* value of less than 0.05 (Wilcoxon). (**E**) GSEA of cluster 0 and (**F**) cluster 2 using Hallmark gene sets. ssGSEA was performed using Escape, and DE gene sets were identified by Wilcoxon test. The top-20 gene sets with an adjusted *P* value of less than 0.05 were plotted, with a positive FC indicating upregulation in pancreatic tissue slice (orange) and negative FC indicating upregulation in pLNs (blue). Up, upregulated.

We next investigated tissue-specific phenotypic differences in these clusters. We noted that CD8^+^ Teff cell clusters 0 and 2, defined by expression of NK cell receptors (*KLRK1* [ref. 66], *KLRB1* [ref. 67]), Th1-related chemokine receptors and ligands (*CXCR3*, *CXCR6*, *CCL5* [ref. 68]), and terminal memory markers (*LAG3*, *KLRD1*, *EOMES* [ref. 69]), were the most commonly shared clusters across tissues, whereas less-differentiated subsets, such as naive T cells, were expectedly more prevalent in pLNs. Additionally, inflammatory macrophages expressing *IL1B* were more prevalent in pancreatic tissues (Figure 5A). Examination of DEGs in total CD8^+^ T cells between tissues revealed increased expression of genes that promote naive/stem-like phenotypes, *LEF1* (log_2_FC = 4.8, *P*_adj_ = 1.6 × 10^–7^) and *SELL* (log_2_FC = 4.0, *P*_adj_ = 1.4 × 10^–7^), in pLNs relative to pancreas (Figure 5B). As this could partially be explained by the increased proportion of naive cells in pLNs, we compared gene expression of shared cell populations across tissues. Cells from cluster 0 (CD8^+^ Teff) derived from pancreatic tissue slices had upregulated markers of cytotoxicity, such as *GZMB* (log_2_FC = 5.46, *P*_adj_ = 1.4 × 10^–46^), and exhaustion, such as *DUSP4* (70) (log_2_FC = 6.6, *P*_adj_ = 1.3 × 10^–57^), alongside downregulation of the common γ chain cytokine receptor genes *IL7R* (log_2_FC = –1.9, *P*_adj_ = 2.5 × 10^–10^) and *IL2RG* (log_2_FC = –2.0, *P*_adj_ = 4.1 × 10^–5^) (Figure 5C). Cluster 2, with a transcriptomic profile similar to that of cluster 0 but with upregulated cytotoxicity and memory markers (CD8^+^ CTLs), demonstrated expression differences similar to those seen in cluster 0 across tissues (Figure 5D). Gene set enrichment analysis (GSEA) of cluster 0 revealed upregulation of inflammatory signaling pathways, namely IL-6/JAK/STAT3 (log_2_FC = 2.3, *P*_adj_ = 1.5 × 10^–19^) and TNF-α signaling (log_2_FC = 0.34, *P*_adj_ = 1.7 × 10^–7^), as well as downregulation of Wnt/β-catenin signaling (log_2_FC = –0.6, *P*_adj_ = 2.3 × 10^–3^) (Figure 5E) in pancreatic tissue slices versus pLNs, altogether suggesting that this cluster acquired a more differentiated transcriptomic program in pancreas relative to pLNs. We observed similar upregulation of inflammatory pathways in cluster 2, representing an activated effector phenotype (Figure 5F). Notably, while Wnt/β-catenin signaling was not significantly enhanced in pLNs for this cluster, we did observe upregulation of *GIMAP5* (71) (log_2_FC = –3.3, *P*_adj_=1.5 × 10^–8^) in pLNs, a gene that is an important negative regulator of GSK3β and thus potentially enhances Wnt signaling. Consistent with prior observations of IL-15 signaling in the pancreas (72), IREA of DEGs from CD8^+^ T cell clusters 0 and 2 between the pLNs and pancreas revealed enriched IL-15 signaling as a driver of differential tissue phenotypes (Supplemental Figure 10).

### CXCR3^+^ naive T cells show features of stemness and polyfunctionality in pLNs.

**Figure 6:**
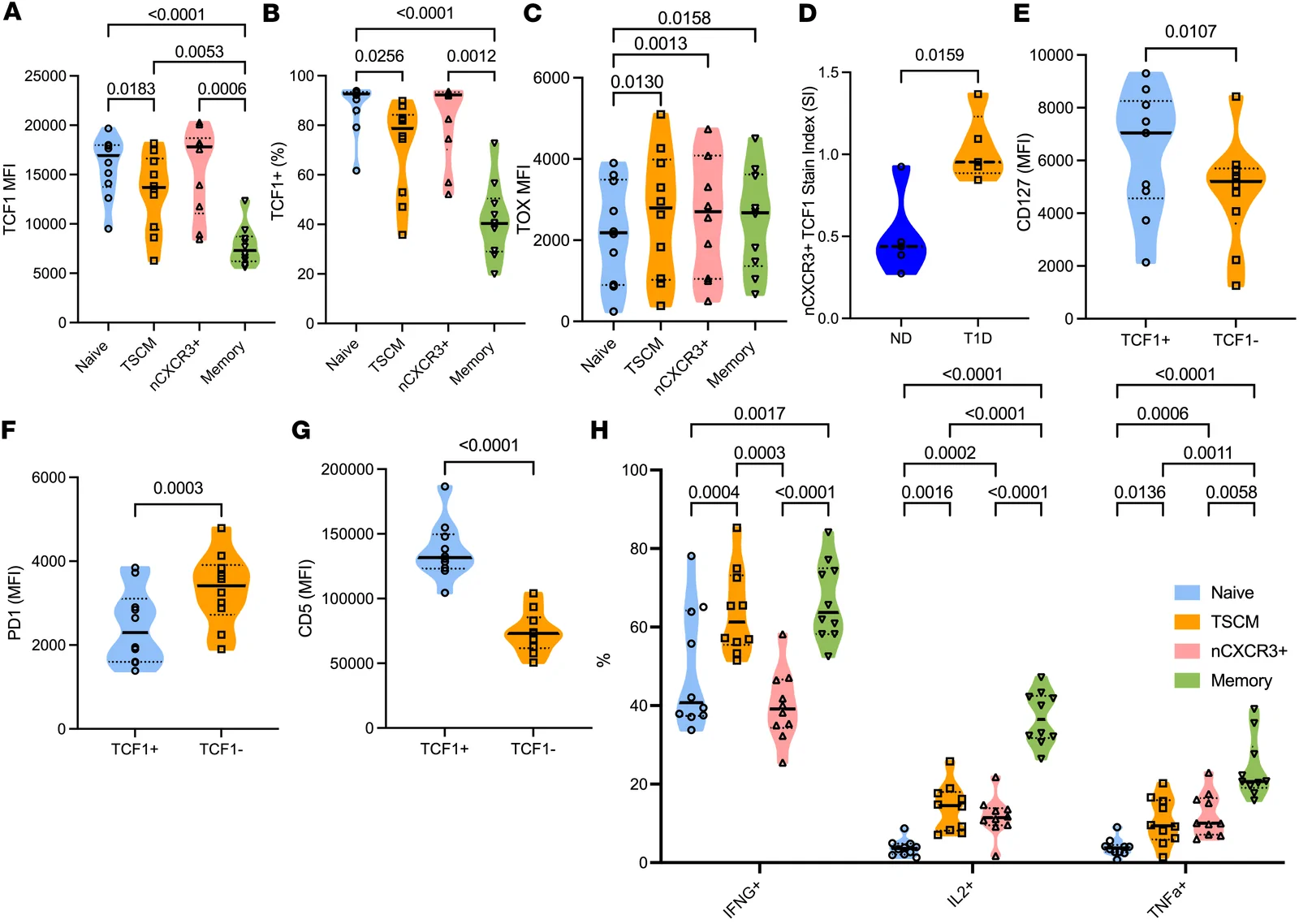
Naive CXCR3^+^CD8^+^ T cells possess features of stemness and polyfunctionality. Flow cytometry–mediated assessment of pLN cells for canonical TSCM markers and cytokine expression. (**A**) Naive CXCR3^+^ (nCXCR3^+^) cells resembled TSCM and naive cells in their expression of TCF1 (*n* = 10; repeated-measures, 1-way ANOVA with Tukey’s multiple-comparison test). (**B**) Enrichment of TCF1^+^ cells in naive CXCR3^+^ cells versus memory cells (*n* = 10; repeated-measures Friedman test with Dunn’s multiple-comparison test). (**C**) TSCM and nCXCR3^+^ cells express more TOX than naive cells (*n* = 10, repeated-measures, 1-way ANOVA with Tukey’s multiple-comparison test). (**D**) T1D pLN nCXCR3^+^ cells express enhanced TCF1, as quantified by the stain index [(median TCF1 expression in nCXCR3^+^ cells – median TCF1 expression in memory CD8^+^ T cells)/(SD of TCF1 in memory CD8^+^ T cells × 2)] (*n* = 5 per group; 2-tailed Mann-Whitney *U* test). (**E**) Upregulation of CD127, (**F**) downregulation of PD-1, and (**G**) upregulation of CD5 in TCF1^+^ versus TCF1^–^ cells (*n* = 10; 2-tailed *t* test). (**H**) After 4 hours, PMA/ionomycin stimulation with GolgiStop, nCXCR3^+^ cells possess more IL-2– and TNF-α–expressing cells than naive cells, more closely resembling TSCMs for expression of these molecules (*n* = 10; repeated-measures, 2-way ANOVA with Tukey’s multiple-comparison test). Data are presented as the median and quartiles.

To better understand the phenotype and function of the naive CXCR3^+^ cells (cluster 0) enriched in T1D pLN, we examined the expression of canonical exhaustion and TSCM markers on pLN CD8^+^ T cells from 5 T1D donors and 5 ND donors by flow cytometry. We confirmed that across all donors, CXCR3^+^ naive CD8^+^ T cells, along with the remainder of the naive cells (CXCR3^–^ naive), expressed significantly more T cell factor 1 (TCF1) (*P* = 0.0006, FC = 2.01) and contained a significantly greater fraction of TCF1^+^ cells than did memory cells (*P* = 0.0012, FC = 1.97) (Figure 6, A and B, and Supplemental Figure 11). We also observed that TSCMs (CD45RA^+^CD45RO^–^CXCR3^–^CD95^+^CD27^+^) and CXCR3^+^ naive CD8^+^ T cells expressed similar levels of TOX, with both populations displaying increased TOX expression relative to CXCR3^–^ naive cells (TSCMs: *P* = 0.0130, FC = 1.21; naive CXCR3^+^: *P* = 0.0013, FC = 1.19) (Figure 6C). Interestingly, we also noted increased TCF1 expression on naive CXCR3^+^ cells from T1D versus ND pLNs (Figure 6D) (*P* = 0.0159, FC=2.08). Supporting the notion that these cells possess a transitional phenotype, TCF1^+^ cells displayed significantly increased CD127 expression (*P* = 0.0107, FC = 1.32), reduced PD-1 expression (*P* = 0.0003, FC = 0.80), and enhanced CD5 expression (*P* < 0.0001, FC = 1.85) as compared with TCF1^–^ cells (Figure 6, E–G). Moreover, after 4 hours of stimulation with PMA and ionomycin, we found that naive CXCR3^+^ cells upregulated expression of IL-2 (*P* = 0.0002, FC = 3.11) and TNF-α (*P* = 0.0006, FC = 3.12) compared with the remainder of the naive (CXCR3^–^) cells (Figure 6H and Supplemental Figure 12), but had reduced IFN-γ expression compared with TSCMs and memory cells, and expression levels of IL-2 and TNF-α similar to those of TSCMs. Importantly, these data support the idea that this subset has features of stemness, polyfunctionality, and a potentially transitional phenotype.

### TCF1^+^TOX^+^ T cells demonstrate tissue-specific phenotypes and localize to islets in human T1D.

**Figure 7:**
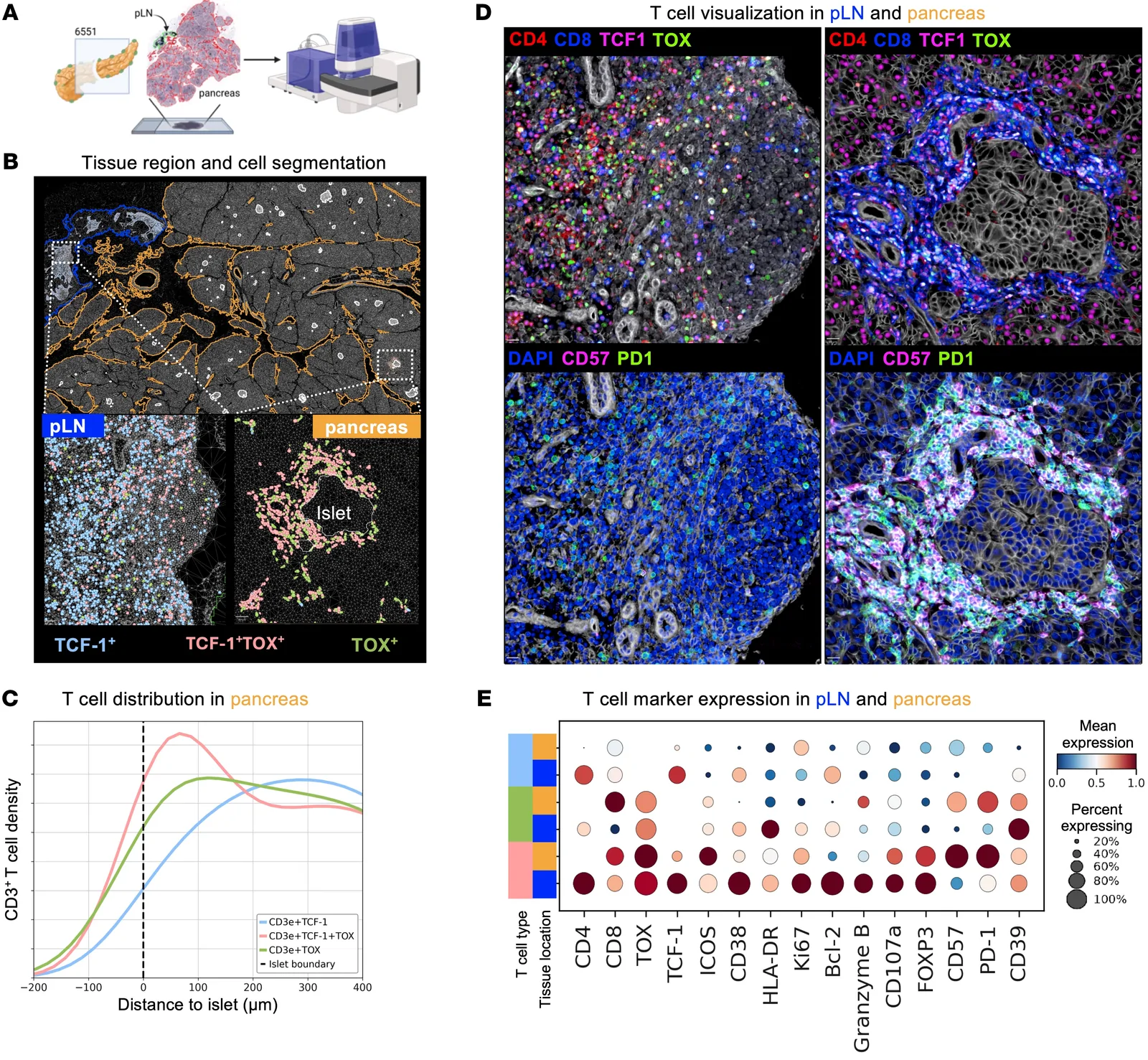
TCF1^+^TOX^+^ cell populations colocalize to the peri-islet area in human T1D. (**A**) Schematic of phenocycler imaging of embedded pLNs and pancreas from nPOD 6551. (**B**) Representative images of pancreas and pLNs, with callouts showing TCF1- and TOX-expressing cell populations. (**C**) Line plot illustrating the density of TCF1- and TOX-expressing T cell populations across islet distance, with TCF1^+^TOX^+^ cells having greater density nearer the islets than single-positive cell populations. (**D**) Representative islet images from donor 6551 showing lineage markers (CD4, CD8) alongside TCF1, TOX, CD57, PD-1, and nuclear stain (DAPI). Original magnification ×20. (**E**) Dot plot showing expression of key memory, negative regulatory, and activation-related markers across TCF1- and TOX-expressing cell populations.

We performed PhenoCycler analysis of pancreas and pLNs from donor 6551 to examine the tissue localization of naive-like and terminal effector phenotypes in insulitis (Figure 7A). CD3^+^ T cells were classified as TCF1^+^TOX^–^ (TCF1^+^, naive), TCF1^–^TOX^+^ (TOX^+^, memory and exhausted), and TCF1^+^TOX^+^ (activated transitional and effector) cell populations (Figure 7B). TCF1^+^TOX^+^ cells in the pancreas were found more often near or within the islet boundary than TCF1^+^ or TOX^+^ cells, with TOX^+^ cells being more proximally located than TCF1^+^ cells (Figure 7C). In accordance with our single-cell analysis of CD8^+^ T cells across tissues, we observed enhanced inhibitory receptor expression (namely, TOX, CD57, PD-1) in TCF1^+^TOX^+^ and TOX^+^ cell subsets in the pancreas relative to pLNs (Figure 7, D and E). Thus, these data indicate a spectrum of memory CD8^+^ T cell phenotypes in the pancreas, which included an activated TCF1^+^TOX^+^ cell population near the islets. Akin to recent reports in NOD mice that precursor diabetogenic T cells are primed and maintained as a self-renewing pool in the pLNs and seed terminally differentiated cells that expand in the islets (5), our data indicate that distinct tissue programs allow for the maintenance of CD8^+^ T cell populations and the prevention of exhaustion in pLNs, as well as acquisition of further effector function in the pancreas during human T1D.

## Discussion

Despite tremendous progress characterizing circulating immune cells in T1D, a critical knowledge gap remains regarding alterations in tissue immune phenotypes. Large pancreas-derived datasets have provided insight into disease-related changes in endocrine composition and function (73, 74), as well as potential changes in immune–islet cell communication (73, 75), but have limited power to examine immune cell phenotypes. Datasets derived from secondary lymphoid organs in T1D are lacking because of limited sample availability and, to date, a paucity of receptor information (76). This study begins to address these gaps using complementary methods (i.e., CyTOF, scRNA-seq/TCR-seq/CITE-seq, spatial proteomics) to assess differences in T cell phenotypes and their potential molecular underpinnings within the pLN and pancreas.

CD8^+^ T cells are classically thought to be a major mediator of islet destruction in T1D, given their abundance in the insulitic lesion (77) and their cytolytic function (78–80). Moreover, recent studies indicate that CD8^+^ T cells with a self-renewing phenotype may be important for T1D pathogenesis, an observation supported by elevations in peripheral blood and phenotypic enrichment in autoreactive cells (5, 14, 81). Our study shows that CD8^+^ T cells expressing TSCM markers (CD45RA, CCR7, CXCR3, CD27, CD28) were increased in T1D pLNs. TSCM cells play an important role in memory maintenance and generate rapidly responding effector cells (82). Serum concentrations of cytokines that promote TSCM development, notably IL-15, are known to be elevated in patients with T1D (72, 83). Moreover, circulating islet antigen–reactive T cells from T1D donors have been shown to possess stem-like features (14). Enhanced capacity for self-renewal could be clinically detrimental, as multiple therapeutic avenues (e.g., teplizumab, anti–thymocyte globulin [ATG]) have converged on a signature of CD8^+^ T cell exhaustion in therapeutic responders (13, 84). Our study provides further support for the relevance of this pathway, as IREA revealed an enrichment of IL-15–induced genes across the trajectory of CD8^+^ T cell differentiation in T1D. Interestingly, pathway analysis of naive cells indicated an enrichment of autophagy and mitophagy in cells from T1D pLNs. IL-15 has recently been shown to enable sustained autophagy during activation compared with inflammatory cytokines (85), potentially better supporting memory cell population maintenance. We also observed an increase in cytokine signaling and upregulation of the antiapoptotic factor *BCL2,* which, coupled with downregulation of multiple exhaustion-associated genes in T1D, could suggest that CD8^+^ Teff cells favor a progenitor exhausted phenotype rather than a terminal exhausted phenotype in T1D (44). Further studies are needed to connect IL-15 signaling in T1D to autoreactive T cell stemness and progenitor exhausted phenotypes, and to determine if inhibition of this pathway aids in establishing durable tolerance. Notably, baricitinib, an inhibitor of JAK1/2 signaling and, thus, common γ chain cytokine signaling (including IL-15), recently demonstrated efficacy in preserving β cell function in donors with recent-onset T1D (86). Interestingly, we observed reduced expression of *IL7R* and *IL2RG* in effector cells derived from the recent-onset T1D pancreas relative to those from paired pLNs, indicating that, in the pancreas microenvironment, these cells may be more reliant on IL-15 and other effector cytokines, such as IL-21, than IL-2 or IL-7. It remains to be seen whether baricitinib alone could aid in targeting the autoreactive T cell reservoir or if combinatorial therapy will be necessary with T cell exhaustion–inducing therapeutics.

Limitations of the study include donor heterogeneity and limited sample size due to the rarity of study tissues. Notably, we were unable to obtain sufficient immune cell numbers from ND pancreatic tissue slices to compare immune phenotypes between T1D and ND donors. And, our small sample size from pancreatic slices was likely not representative of the full complement of immune cell phenotypes present. Our laboratory is currently utilizing spatial transcriptomics (87) and proteomics technologies to assess this without the need for immune cell isolation. Moreover, T1D is a heterogeneous disease, and it is possible that immune phenotypes identified through our studies may differ across different age or ancestry groups, which our study was not powered to assess. Despite this, we believe the information we have provided using paired phenotype and receptor information for these subsets can be used by the field to identify CDR3 motifs of further interest.

As it stands, known TCR sequences that correspond to autoreactive T cell specificity are rare (60). Moreover, matching sequences to those in curated databases such as the Manually curated database of Pathology Associated Sequences (McPAS) (88) and the Immune Epitope Database (IEDB) (89) introduces a bias toward specificities and epitopes of interest. Indeed, we were unable to confidently identify a priori T1D antigen autoreactive cells using peptide-MHC (pMHC) dextramer reagents. This could reflect low sampling (*n* = 10,000 cells per donor), incomplete knowledge of antigen reactivity, and/or individual heterogeneity in autoantigen reactivity. However, utilizing a list of experimentally validated T1D clones, we identified 11 putative T1D clones in the pLNs. Nevertheless, there is a need to apply new analytical strategies to identify similarities in TCR sequence and transcriptomic features that may indicate a common antigen specificity (90). We utilized one such tool, CoNGA (57), to examine joint gene expression and receptor features in T1D. This analysis revealed a T1D-associated enrichment of a TCR cluster composed of diverse naive CD8^+^ T cells that displayed features of prior activation (i.e., *KLRK1*, *HCST*, low *CXCR3*), further supporting our gene expression and proteomics data. The feature of low AF4 within this cluster supports previous data in which autoreactive T cells were found to possess more hydrophobic residues (91), including within autoreactive receptors detected in the pancreas (61). Moreover, in T1D pLNs, we also found enrichment of a more terminally differentiated cluster of CD8^+^ T cells, which displayed expression of cytotoxicity-related genes, elevated *CXCR3*, and the memory/exhaustion–related gene *TOX*, while retaining some *TCF7* expression. Although infrequent, we postulated that this cell population may represent a transitional memory phenotype that is poised to infiltrate the pancreas, as supported by the presence of an expanded putative autoreactive clone in this population in a donor with new-onset T1D.

We utilized paired pLN and pancreas T cell data to investigate shared phenotypes and to interrogate whether local expansion of T cells occurred in fresh pancreatic slices. We identified the most abundant and clonally expanded shared populations to be effector CD8^+^ T cells. Moreover, we noted total CD8^+^ T cells had reduced expression of genes involved in promoting stem-like/naive phenotypes (*LEF1*, *SELL*) in pancreas compared with pLNs. This could, in part, be driven by the local inflammatory milieu. Indeed, we observed enrichment of multiple inflammatory cytokine signaling pathways, namely IL-6 and TNF-α, in pancreas relative to pLN T cells, while we observed upregulation of Myc, mTORC1, and Wnt signaling pathways in pLNs relative to pancreatic T cells. While inter-donor sharing of TCR clones was limited and difficult to detect due to the sparsity of single-cell data, we were able to detect intra-donor sharing of clones across tissues and between clusters. Donor 6551, a 20-year-old donor with 0.58 years’ T1D duration, particularly notable for insulitis severity, had expanded CD8^+^ Teff clones that were shared between tissues and between clusters. In the donor with longer-duration (4 years) T1D with less insulitis (donor 6536), we were able to detect sharing of only 1 effector CD8^+^ clone across clusters and 1 CD4^+^ clone across tissues.

Using PhenoCycler analysis, we were able to show that in the same T1D donor with marked insulitis, donor 6551, TCF1- and TOX-expressing T cell populations can be found in pLNs and pancreas. Importantly, we found that TCF1^+^TOX^+^ cells possessed an enhanced terminal effector phenotype in the pancreas and could be found proximal to the islets. We also found by flow cytometry that pLN naive CXCR3^+^ T cells had expression levels of the transcription factors TCF1 and TOX similar to those of TSCMs and produced similar levels of proinflammatory cytokines upon activation. Akin to human peripheral blood data, we noted that TCF1-expressing cell populations in the pLNs tended to express less PD-1 and more CD127 (92) and CD5 (93), indicative of an early differentiation stage and activation potential. In accordance with murine studies (5), these data collectively support the notion that T cell clones are supplied from the pLNs and adopt a more effector and terminally differentiated phenotype in the pancreas during human T1D development. Previously, our laboratory reported increased circulating CXCR3^lo^ CD8^+^ T cells in T1D (20) following phenotyping of a cohort of over 824 cross-sectional peripheral blood samples. The present study supports a role for these cells in the draining LN: a small set of related terminal effector CXCR3-expressing cells could be found in the pLNs and a larger fraction in the target organ, suggesting that these cells share some features of stemness (TCF1 expression) and are more polyfunctional as compared with naive cells. Identifying subsets of interest that are relevant in both the peripheral blood and the target organ could aid efforts to selectively inhibit autoreactivity and monitor outcomes. Importantly, combination CXCR3 antagonism with ACT-777991 and anti-CD3 treatment leads to disease remission in the NOD mouse (94), with first-in-human trials in healthy adults showing that this compound is tolerable (95). Thus, combinatorial strategies to induce tolerogenic phenotypes while targeting pathways relevant to tissue immune phenotypes may represent an avenue to achieve durable tolerance. Taken together, our studies indicate that memory and effector T cell programs in T1D were maintained in the pLNs and enhanced locally in the pancreas, and that this was likely driven by aberrant regulation of cytokine signaling and affected by genotype at T1D risk loci. Future directions validating the functionality of the clones identified here and investigating the cellular interactions of CXCR3^+^CD8^+^ T cells across the differentiation trajectory will inform the development of therapeutics to modulate these clones and subsets, providing novel avenues to delay or prevent T1D progression.

## Methods

### Sex as a biological variable.

Male and female donors were accepted in this study. Sex was added as a latent variable in single cell differential gene expression analyses of pLN to help control for potential sex-based gene expression differences.

### Human organ donors.

Tissues were recovered from donors with T1D, AAb^+^, and ND donors according to the nPOD inclusion criteria (96). Demographic information used to stratify individuals for analyses was obtained from the nPOD datashare (https://npoddatashare.coh.org/) and the nPOD data portal (https://portal.jdrfnpod.org). Supplemental Table 6 describes the cases and corresponding experiment information.

### Organ processing.

Tissues were processed as previously described (16). Cells were freshly analyzed by flow cytometry as described below, and remaining cells were cryopreserved using CryoStor CS10 (STEMCELL Technologies). Live pancreatic tissue slices were prepared from nPOD donors as previously described (97) and freshly digested with 1× collagenase IV (STEMCELL Technologies) diluted in DMEM for 30 minutes at 37^o^C and 5% CO_2_. The remaining tissue was gently mechanically dissociated with frosted slides and filtered using 70 μm filters (Miltenyi Biotec) to obtain a single-cell suspension. Cells were enriched for CD45^+^ cells using CD45 Microbeads (Miltenyi Biotec) and processed for scRNA-seq (10X Genomics) as described below.

### Flow cytometry.

Fresh tissues were dissociated as described above, stained with Live/Dead yellow viability dye (Invitrogen, Thermo Fisher Scientific), and incubated with TruStain FcX (BioLegend) according to the manufacturer’s instructions before staining with an antibody panel broadly examining memory, effector, and naive immune cell phenotypes (Supplemental Table 7), as previously reported (20). Data were acquired on a BD LSRFortessa. To assess expression of transcription factors in CD8^+^ T cell populations, cryopreserved pLN cell suspensions were thawed, stained with Zombie Aqua viability dye (Invitrogen, Thermo Fisher Scientific), incubated with TruStain FcX, and stained with antibodies (Supplemental Table 8). After extracellular staining, cells were washed twice with stain buffer (PBS, 2% FBS, 0.05% NaN3) and then fixed and permeabilized using the True Nuclear Transcription Factor Buffer Set (BioLegend) according to the manufacturer’s instructions. Cells were blocked with normal rat serum before staining for transcription factors overnight at 4°C in permeabilization buffer. Cells were washed with stain buffer before reading on a Cytek Aurora 5L cytometer. For analysis of cytokine expression, cryopreserved pLN suspensions were thawed and incubated at a concentration of 1 × 10^6^/mL with Cell Stimulation Cocktail (Invitrogen, Thermo Fisher Scientific) in the presence of 0.66 μL/mL BD GolgiStop for 4 hours. Cells were stained with Zombie Aqua (Invitrogen, Thermo Fisher Scientific), incubated with TruStain FcX, and stained with an antibody panel (Supplemental Table 9). After extracellular staining, cells were fixed with BD Cytofix and permeabilized with Intracellular Staining Perm Wash Buffer (BioLegend). Staining for cytokines was conducted overnight at 4°C in Perm Wash Buffer. Cells were washed with Perm Wash Buffer prior to reading on a Cytek Aurora 5L cytometer. Data were analyzed using FlowJo software, version 10.8.2.

### Mass cytometry.

Cryopreserved cells from pLNs of ND donors (*n* = 12) and T1D donors (*n* = 10) were stained with a 35-marker panel (Supplemental Table 10) of MAXPAR metal-chelating polymers and run on a CyTOF mass cytometer by the Human Immune Monitoring Center at Stanford University, as reported previously (98).

### Mass cytometric analysis.

Data were normalized between batches using premessa (99) in R, based on internal bead controls. Next, flow cytometry standard (FCS) files were loaded into FlowJo (BD Biosciences), beads were excluded, and live single CD45^+^ cells were used for downstream analysis (Supplemental Figure 13). Data analysis involved importing FCS files into R and creating a flowset using the flowCore package (100). Data were arcsinh transformed with a cofactor of 5 before using the expression data and cell metadata to create a Seurat object. Principal component analysis (PCA) was run using all panel markers, after which *k*-nearest-neighbors (*k*-NNs) were calculated using FindNeighbors (), and initial clustering using the Louvain algorithm was performed (resolution = 0.2). PCA was re-run on subsetted data, with markers not associated with the lineage of interest removed. Nearest neighbors were calculated as above, and subclustering was performed as above for each subset using the Louvain algorithm (resolution = 0.2). Cell counts per cluster per individual were normalized by the total number of cells to allow for comparison of cluster proportions. Differential abundance analysis was performed using propeller (101), with age and batch as covariates in a linear model designed with model.matrix (~0 + group + age + batch). Design matrix and contrasts (T1D-ND) were incorporated into the propeller.ttest function with the arguments robust=T, trend=F, and sort=T. Significantly altered cluster proportions were defined as a Bonferroni-adjusted *P* value of less than 0.05.

### scRNA-seq.

Cryopreserved single-cell suspensions were thawed and stained with a cocktail of 7 oligonucleotide-barcoded TotalSeq antibodies (BioLegend) (Supplemental Table 11) and peptide-MHC dextramers (Immudex) (Supplemental Table 11) to aid clustering naive and memory CD8^+^ and CD4^+^ T cell phenotypes and identification of antigen-specific cells. Prior to use, Totalseq antibodies were centrifuged at 14,000*g* for 10 minutes at 4°C to avoid antibody aggregates. Cell viability was assessed using acridine orange and propidium iodide (AO/PI) staining on a Nexcelom Cellometer or CellDrop. For 6 samples with viability of less than 70%, the Dead Cell Removal Kit (STEMCELL Technologies) was used. Cells (1 × 10^6^ per sample; viability >70%) were resuspended in 100 μL PBS plus 1% BSA before incubation for 10 minutes at room temperature with 2 μL of each pMHC dextramer, according to the manufacturer’s instructions, followed by incubation with TruStain FcX (5 μL/test) to block Fc receptors for 10 minutes at 4°C, and incubation with the antibody master mix (0.5 μL of each antibody/test) for 30 minutes at 4°C. Cells were washed 4 times with 3 mL chilled PBS plus 1% BSA, after which live cells were counted using AO/PI on a Nexcelom Cellometer or CellDrop, and volumes were adjusted to obtain a targeted recovery of 10,000 cells. Single-cell suspensions were loaded into the Chromium Controller (10X Genomics), and libraries were prepared according to 5′ version 1.1, 5′ version 2, and 5′ HT version 2 kit protocols (10X Genomics). Libraries were sequenced on an Illumina NovaSeq or a NovaSeq X instrument with a target of 50,000 paired reads for gene expression, 10,000 paired reads for surface protein, and 5,000 paired reads for TCR libraries. Median gene expression library sequencing saturation was 80.9%.

### Single-cell read processing.

Binary base call files were processed to fastq files using the cellranger mkfastq pipeline (10X Genomics). Fastq files from gene expression, surface protein, and TCR libraries were processed to matrices using the cellranger multi (10X Genomics) count pipeline to maximize counting of cells with all 3 modalities.

### scRNA-seq normalization and analysis pipeline.

scRNA-seq data were subjected to QC by removing doublets using doubletfinder (102), and cells were removed according to cutoffs of reads/cell of 2,000 or fewer and percentage of mitochondrial reads of 10% or less, and then normalized using denoised and scaled by background (103) to remove background and ambient signal from RNA and protein libraries. Data were log-normalized with NormalizeData () and scaled with ScaleData (), and then variable features were calculated, and dimensionality reduction was performed in Seurat (104). Lack of observed pMHC dextramer signal led to exclusion from future analysis. For antibody-derived tag data, variable features were set to all antibodies included, data were scaled (104), and PCA was run. Data were integrated in Seurat using IntegrateLayers () with reciprocal PCA (RPCA) as the integration method. After integration of gene expression data by patient ID, no significant batch effects were observed (Supplemental Figure 14). After subsetting T cell clusters, data were re-normalized, PCA was re-run, and RPCA integration with IntegrateLayers () was run again on this subset. Integration of TCR with gene expression data was performed using scRepertoire (105) in R. For pancreas and pLN clone comparisons, clones were defined as sharing an identical CDR3 amino acid sequence for both TCRα and TCRβ. DEGs in pLNs were calculated using MAST (106), with sex as a latent variable with a minimum expression threshold of 0.10 and reported when the FDR-corrected *P* value was less than 0.05 and the log_2_FC was 0.26 or greater. DEGs between pLN and pancreatic slice clusters were calculated using the Wilcoxon rank-sum test with a minimum expression threshold of 0.10 and reported when the FDR-corrected *P* value was less than 0.05 and the log_2_FC was 0.26 or greater. Pathway enrichment analyses were performed using the top 100 most DEGs (other than for the exhausted CD8^+^ T cell cluster and the Tcm cluster, for which only 53 and 86 genes met our significance thresholds, respectively) between T1D and ND donors, with the FDR *P* of less than 0.05 per cluster ranked by log_2_FC, as input into the enrichPathway function in the ReactomePA (107) package, for pLNs. The R package escape (108) was used to perform single-sample GSEA (ssGSEA) with the integrated pancreatic slice and pLN dataset and normalized with nFeature RNA as the scale factor. Results were reported when the Benjamini-Hochberg–corrected *P* value was less than 0.1, as noted in the figure legends. IREA was performed by inputting the top (*P* < 0.05, log_2_FC >0.26) DEGs between T1D and ND donors per cluster using the IREA web tool (https://www.immune-dictionary.org/app/home) with cell type “T_cell_CD8,” species “Human,” and Method parameter “Score.”

### CoNGA.

The RNA assay from the integrated pLN Seurat object was exported in h5ad format for Python. Filtered contig annotation data from each sample were merged using make_10x_clones_file_batch from the conga package in Python. The resulting merged TCR data were used along with the gene expression data as input for run_conga.py (57) with the –all flag and batch keys set to status and nPOD identification number (ID) to compare the composition of clusters by clinical status and across all donors. Gene expression data were also batch corrected using the argument batch_integration_method harmony with batch_integration_key batch. For CoNGA, the cellranger VDJ clonotype definition of identical CDR3 nucleotide sequence was used. Clones that possessed a conga score below 1, indicative of significant overlap between gene expression and TCR neighborhoods beyond what would be expected by chance (57), were used as input to evaluate enrichment in cluster composition by clinical status using a 2-sided Fisher’s exact test.

### PhenoCycler multiplex imaging.

Immunofluorescence of pancreas with embedded pLNs was performed using the PhenoCycler-Fusion System (Akoya Biosciences/Quanterix) according to the manufacturer’s recommendation for formalin-fixed, paraffin-embedded (FFPE) tissue. Deparaffinization was performed by baking at 60°C followed by xylene washes. Tissue rehydration was accomplished through graded ethanol/DI-H_2_O slide washes (2 times at 100%, 90%, 70%, 50%, and 30%, 2 times with DI-H_2_O). Antigen retrieval was performed by immersing sections in 1× antigen retrieval buffer under high pressure in a pressure cooker. Tissue was washed in hydration buffer followed by then staining buffer. Primary antibody staining was performed at room temperature using 175 μL antibody cocktail (Supplemental Table 12) (IO60 panel, catalog PDPIO60H), blocking reagents (N, G-v3, J, S), and blocking buffer. Tissue was rinsed in staining buffer and sequentially postfixed, followed by PBS washes in 1.6% paraformaldehyde, then ice-cold methanol, and then fixative solution. Tissue was kept overnight in storage buffer at 4°C. A 96-well reporter plate was prepared with either cycle-specific cocktails of complementary oligonucleotide-conjugated fluorescent secondary antibodies, nuclear stain (DAPI), and reporter stock solution for cyclic protein detection or only reporter stock solution for blank cycle autofluorescence detection. A flow cell was press-sealed to the face of the slide for microfluidic delivery of reagents.

Automated detection and imaging were performed by first generating an experiment file specifying target-barcode pairs and exposure times for each cycle (Experiment Designer software, Akoya Biosciences/Quanterix). PhenoCycler Buffer and dimethyl sulfoxide solutions (20% and 90% in 1× PhenoCycler Buffer) were prepared and loaded in the PhenoCycler-Fusion instrument bottles. Following automated calibration and equipment checks, tissue was autodetected, and images were acquired with 16 bit precision at ×20 magnification, automatically processed for stitching and autofluorescence subtraction, and saved in the proprietary “qptiff” format.

### Phenocycler data analysis.

Semantic segmentation was performed in QuPath (109), version 6.0 T, on the qptiff file for donor 6551. Objects (pLN/pancreatic tissue area and islets) were classified using the pixel classifier with default settings. Cell and nuclear boundaries were generated with the Instanseg (110), version 0.1.5, extension’s default model “fluorescence_nuclei_and_cells-0.1.1” using all channels as input, default cell measurements, and default outputs. Statistics for each cell’s shape and marker intensity, as well as centroid coordinates and signed distance to the nearest islet, were exported as a .csv file for analysis in a custom Python Jupyter notebook (111).

### Statistics.

Data were analyzed using R version 4.2.3, Python version 3.9, and GraphPad Prism version 9 (GraphPad Software). Statistical tests used to determine significance include linear and generalized linear models, Wilcoxon rank-sum test, 2-way Fisher’s exact test, 1- and 2-way ANOVAs with Tukey’s multiple-comparison test, Friedman test with Dunn’s multiple comparison test, and 2-tailed *t* tests and Mann-Whitney U tests. Unless otherwise noted, *P* values of less than 0.05 were considered significant. Data are presented in the figure legends as mean ± SEM and median and quartiles.

### Study approval.

The study was conducted in accordance with federal guidelines, and the University of Florida (UF) IRB approved protocol IRB201600029, as described previously (112).

### Data and code availability.

Mass cytometric and flow cytometric data are available at ImmPort (https://www.immport.org, accession number: SDY3066). Supporting data values associated with this manuscript are provided in the Supporting Data Values file. Gene expression and TCR data are available in the Gene Expression Omnibus (GEO) database (GSE298811). Phenocycler data are available upon request, and code used for Phenocycler analysis is accessible at https://github.com/smith6jt-cop/Panc_pLN_Analysis

## Author contributions

LDP Designed research studies, conducted experiments, acquired data, analyzed data, wrote the original draft of the manuscript, and acquired funding. HRS conducted experiments, acquired data, and reviewed and edited the manuscript. JAS conducted experiments, acquired data, analyzed data, and wrote the original draft of the manuscript. ALP reviewed and edited the manuscript. RLB acquired data and reviewed and edited the manuscript. CHW reviewed and edited the manuscript. MAA reviewed and edited the manuscript and acquired funding. RB analyzed data, reviewed and edited the manuscript, and interpreted data. MAB designed research studies, interpreted data, reviewed and edited the manuscript, and oversaw the project. TMB designed research studies, interpreted data, reviewed and edited the manuscript, acquired funding, and oversaw the project.

## Conflict of interest

The authors have declared that no conflict of interest exists.

## Funding support

This work is the result of NIH funding, in whole or in part, and is subject to the NIH Public Access Policy. Through acceptance of this federal funding, the NIH has the right to make the work publicly available in PubMed Central. The content and views expressed are the responsibility of the authors and do not necessarily reflect the official view of the nPOD (RRID: SCR_014641).

- NIH (P01 AI042288, to TMB; F31 DK129004, and 5T32DK108736, to LDP).
- American Diabetes Association (ADA) (11-23-PDF-78, to LDP).
- The Leona M. and Harry B. Helmsley Charitable Trust (2301-06562, to TMB).
- Breakthrough T1D and The Leona M. & Harry B. Helmsley Charitable Trust (3-SRA-2023-1417-S-B, to MAA).

## Supplementary Material

Supplemental data

Supporting data values

## Figures and Tables

**Table 1:**
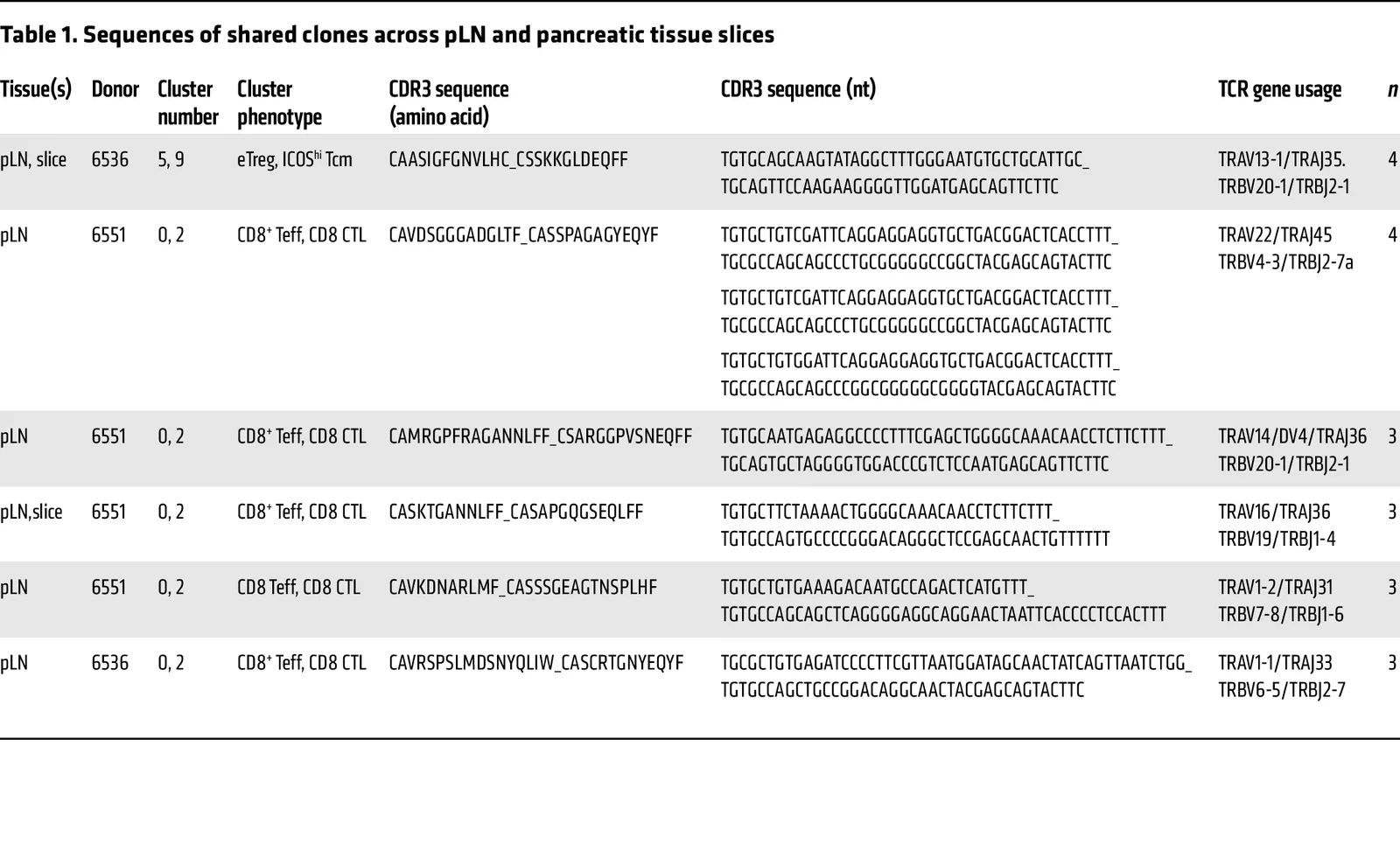
Sequences of shared clones across pLN and pancreatic tissue slices

## Version 1 09/15/2026

Electronic publication

## References

[1] Onengut-Gumuscu S (2015). Fine mapping of type 1 diabetes susceptibility loci and evidence for colocalization of causal variants with lymphoid gene enhancers. Nat Genet.

[2] Wan X (2018). Pancreatic islets communicate with lymphoid tissues via exocytosis of insulin peptides. Nature.

[3] Turley S (2003). Physiological beta cell death triggers priming of self-reactive T cells by dendritic cells in a type-1 diabetes model. J Exp Med.

[4] Marrero I (2016). High-throughput sequencing reveals restricted TCR Vβ usage and public TCRβ clonotypes among pancreatic lymph node memory CD4(+) T cells and their involvement in autoimmune diabetes. Mol Immunol.

[5] Gearty SV (2022). An autoimmune stem-like CD8^+^ T cell population drives type 1 diabetes. Nature.

[6] Vecchione A (2021). Reduced follicular regulatory t cells in spleen and pancreatic lymph nodes of patients with type 1 diabetes. Diabetes.

[7] Ferraro A (2011). Expansion of Th17 cells and functional defects in T regulatory cells are key features of the pancreatic lymph nodes in patients with type 1 diabetes. Diabetes.

[8] Putnam AL (2009). Expansion of human regulatory T-cells from patients with type 1 diabetes. Diabetes.

[9] McClymont SA (2011). Plasticity of human regulatory T cells in healthy subjects and patients with type 1 diabetes. J Immunol.

[10] Sebastiani G (2017). Regulatory T-cells from pancreatic lymphnodes of patients with type-1 diabetes express increased levels of microRNA miR-125a-5p that limits CCR2 expression. Sci Rep.

[11] Arif S (2014). Erratum. Blood and islet phenotypes indicate immunological heterogeneity in type 1 diabetes. Diabetes 2014;63:3835–3845. Diabetes.

[12] Diggins KE (2021). Exhausted-like CD8+ T cell phenotypes linked to C-peptide preservation in alefacept-treated T1D subjects. JCI Insight.

[13] Long SA (2016). Partial exhaustion of CD8 T cells and clinical response to teplizumab in new-onset type 1 diabetes. Sci Immunol.

[14] Abdelsamed HA (2020). Beta cell-specific CD8+ T cells maintain stem cell memory-associated epigenetic programs during type 1 diabetes. Nat Immunol.

[15] Balmas E (2023). Islet-autoreactive CD4+ T cells are linked with response to alefacept in type 1 diabetes. JCI Insight.

[16] Seay HR (2016). Tissue distribution and clonal diversity of the T and B cell repertoire in type 1 diabetes. JCI Insight.

[17] Wiedeman AE (2020). Autoreactive CD8+ T cell exhaustion distinguishes subjects with slow type 1 diabetes progression. J Clin Invest.

[18] Laban S (2018). Heterogeneity of circulating CD8 T-cells specific to islet, neo-antigen and virus in patients with type 1 diabetes mellitus. PLoS One.

[19] Uno S (2010). Expression of chemokines, CXC chemokine ligand 10(CXCL10) and CXCR3 in the inflamed islets of patients with recent-onset autoimmune type 1 diabetes. Endocr J.

[20] Shapiro MR (2023). Human immune phenotyping reveals accelerated aging in type 1 diabetes. JCI Insight.

[21] Cao C (2023). Case Report: A novel mutation in TNFAIP3 in a patient with type 1 diabetes mellitus and haploinsufficiency of A20. Front Endocrinol(Lausanne).

[22] Meixner A (2004). JunD regulates lymphocyte proliferation and T helper cell cytokine expression. EMBO J.

[23] Bedford JG (2019). Rapid IFN independent expression of IFITM3 following T cell activation protects cells from influenza virus infection. PLoS One.

[24] Merkley SD (2018). Modulating T cell responses via autophagy: the intrinsic influence controlling the function of both antigen-presenting cells and T cells. Front Immunol.

[25] Kumar AV (2022). Selective autophagy receptor p62/SQSTM1, a pivotal player in stress and aging. Front Cell Dev Biol.

[26] Zheng MM (2025). IMPDH inhibitors upregulate PD-L1 in cancer cells without impairing immune checkpoint inhibitor efficacy. Acta Pharmacol Sin.

[27] Baldwin JG (2025). Restoring the supply lines: removing roadblocks to fatty acid uptake enhances T cell-driven cancer fight. Signal Transduct Target Ther.

[28] Kolumam GA (2005). Type I IFNs act directly on CD8 T cells to allow clonal expansion and memory formation in response to viral infection. J Exp Med.

[29] Ng D (2015). A lymphotoxin/type I IFN axis programs CD8+ T cells to infiltrate a self-tissue and propagate immunopathology. J Immunol.

[30] Carow B, Rottenberg ME (2014). SOCS3, a major regulator of infection and inflammation. Front Immunol.

[31] Joosten LA (2006). IL-32, a proinflammatory cytokine in rheumatoid arthritis. Proc Natl Acad Sci U S A.

[32] Ciecko AE (2019). Interleukin-27 is essential for type 1 diabetes development and Sjögren syndrome-like inflammation. Cell Rep.

[33] Jaiswal H (2021). The NF-κB regulator Bcl-3 restricts terminal differentiation and promotes memory cell formation of CD8+ T cells during viral infection. PLoS Pathog.

[34] Sun Q (2023). BCL6 promotes a stem-like CD8(+) T cell program in cancer via antagonizing BLIMP1. Sci Immunol.

[35] Seo H (2021). BATF and IRF4 cooperate to counter exhaustion in tumor-infiltrating CAR T cells. Nat Immunol.

[36] Ilangumaran S (2017). SOCS1: regulator of T cells in autoimmunity and cancer. Curr Top Microbiol Immunol.

[37] Lu D (2020). The phosphatase PAC1 acts as a T cell suppressor and attenuates host antitumor immunity. Nat Immunol.

[38] Srirat T (2024). NR4a1/2 deletion promotes accumulation of TCF1(+) stem-like precursors of exhausted CD8(+) T cells in the tumor microenvironment. Cell Rep.

[39] Yang H (2019). Stress-glucocorticoid-TSC22D3 axis compromises therapy-induced antitumor immunity. Nat Med.

[40] Cantor J (2011). Loss of T cell CD98hc specifically ablates T cell clonal expansion and protects from autoimmunity. J Immunol.

[41] Ng SS (2020). The NK cell granule protein NKG7 regulates cytotoxic granule exocytosis and inflammation. Nat Immunol.

[42] Li D (2015). Intermediate Filament(IF) protein vimentin regulates T cell mediated immune response in Gvhd. Blood.

[43] Kollis PM (2022). Characterising distinct migratory profiles of infiltrating T-cell subsets in human glioblastoma. Front Immunol.

[44] Nagasaki J (2024). T cells with high BCL-2expression induced by venetoclax impact anti-leukemic immunity “graft-versus-leukemia effects”. Blood Cancer J.

[45] Kurtulus S (2011). Bcl-2 allows effector and memory CD8+ T cells to tolerate higher expression of Bim. J Immunol.

[46] Cao C (2024). CXCR4 orchestrates the TOX-programmed exhausted phenotype of CD8(+) T cells via JAK2/STAT3 pathway. Cell Genom.

[47] Donato R (1999). Functional roles of S100 proteins, calcium-binding proteins of the EF-hand type. Biochim Biophys Acta.

[48] Ganes S, Fensterl V (2015). IFN-induced Ifit proteins: their role in viral pathogenesis. J Virol.

[49] Su L (2014). HMGN2, a new anti-tumor effector molecule of CD8(+) T cells. Mol Cancer.

[50] Wang Y (2021). HLA-DR expression level in CD8(+) T cells correlates with the severity of children with acute infectious mononucleosis. Front Immunol.

[51] Lyngstadaas AV (2021). Pro-resolving mediator annexin a1 regulates intracellular Ca(2+) and mucin secretion in cultured goblet cells suggesting a new use in inflammatory conjunctival diseases. Front Immunol.

[52] Momenilandi M (2024). FLT3L governs the development of partially overlapping hematopoietic lineages in humans and mice. Cell.

[53] Brault L (2012). PIM kinases are progression markers and emerging therapeutic targets in diffuse large B-cell lymphoma. Br J Cancer.

[54] Cui A (2024). Dictionary of immune responses to cytokines at single-cell resolution. Nature.

[55] Lee J (2023). IL-15 promotes self-renewal of progenitor exhausted CD8 T cells during persistent antigenic stimulation. Front Immunol.

[56] Klebanoff CA (2004). IL-15 enhances the in vivo antitumor activity of tumor-reactive CD8+ T cells. Proc Natl Acad Sci U S A.

[57] Schattgen SA (2022). Integrating T cell receptor sequences and transcriptional profiles by clonotype neighbor graph analysis(CoNGA). Nat Biotechnol.

[58] Ogasawara K (2004). NKG2D blockade prevents autoimmune diabetes in NOD mice. Immunity.

[59] Li S (2022). DAP10 integration in CAR-T cells enhances the killing of heterogeneous tumors by harnessing endogenous NKG2D. Mol Ther Oncolytics.

[60] Mitchell AM (2023). Tracking DNA-based antigen-specific T cell receptors during progression to type 1 diabetes. Sci Adv.

[61] Linsley PS (2024). Germline-like TCR-α chains shared between autoreactive T cells in blood and pancreas. Nat Commun.

[62] Anderson AM (2021). Human islet T cells are highly reactive to preproinsulin in type 1 diabetes. Proc Natl Acad Sci U S A.

[63] Liang T (2017). human pancreatic slice preparations offer a valuable model for studying pancreatic exocrine biology. J Biol Chem.

[64] Marciniak A (2014). Using pancreas tissue slices for in situ studies of islet of Langerhans and acinar cell biology. Nat Protoc.

[65] Huber MK (2025). Beta cell dysfunction occurs independently of insulitis in type 1 diabetes pathogenesis. Cell Rep.

[66] Sutherland CL (2002). UL16-binding proteins, novel MHC class I-related proteins, bind to NKG2D and activate multiple signaling pathways in primary NK cells. J Immunol.

[67] Kirkham CL, Carlyle JR (2014). Complexity and diversity of the NKR-P1:Clr(Klrb1:Clec2) recognition systems. Front Immunol.

[68] Yoon SH (2009). Selective addition of CXCR3(+) CCR4(-) CD4(+) Th1 cells enhances generation of cytotoxic T cells by dendritic cells in vitro. Exp Mol Med.

[69] Wang Y (2023). Characteristics of premanufacture CD8(+)T cells determine CAR-T efficacy in patients with diffuse large B-cell lymphoma. Signal Transduct Target Ther.

[70] Zhao Y (2023). An integrative analysis of the single-cell transcriptome identifies DUSP4 as an exhaustion-associated gene in tumor-infiltrating CD8^+^ T cells. Funct Integr Genomic.

[71] Patterson AR (2018). Gimap5-dependent inactivation of GSK3β is required for CD4 T cell homeostasis and prevention of immune pathology. J Immunol.

[72] Chen J (2013). Insulin-dependent diabetes induced by pancreatic beta cell expression of IL-15 and IL-15Rα. Proc Natl Acad Sci U S A.

[73] Wang YJ (2019). Multiplexed in situ imaging mass cytometry analysis of the human endocrine pancreas and immune system in type 1 diabetes. Cell Metab.

[74] Ackermann AM (2016). Integration of ATAC-seq and RNA-seq identifies human alpha cell and beta cell signature genes. Mol Metab.

[75] Fasolino M (2022). Single-cell multi-omics analysis of human pancreatic islets reveals novel cellular states in type 1 diabetes. Nat Metab.

[76] Golden GJ (2024). Immune perturbations in human pancreas lymphatic tissues prior to and after type 1 diabetes onset. Nat Commun.

[77] Coppieters KT (2012). Demonstration of islet-autoreactive CD8 T cells in insulitic lesions from recent onset and long-term type 1 diabetes patients. J Exp Med.

[78] Dean JW (2020). Innate inflammation drives NK cell activation to impair Treg activity. J Autoimmun.

[79] Peters LD (2023). Modeling cell-mediated immunity in human type 1 diabetes by engineering autoreactive CD8(+) T cells. Front Immunol.

[80] Newby BN (2017). Type 1 IFNs potentiate human CD8^+^ t-cell cytotoxicity through a stat4-and granzyme b-dependent pathway. Diabetes.

[81] Vignali D (2018). Detection and characterization of CD8+ autoreactive memory Stem T cells in patients with type 1 diabetes. Diabetes.

[82] Wang YT (2021). Stem cell-like memory T cells: The generation and application. J Leukoc Biol.

[83] Kuczynski S (2005). IL-15 is elevated in serum patients with type 1 diabetes mellitus. Diabetes Res Clin Pract.

[84] Jacobsen LM (2023). Responders to low-dose ATG induce CD4+ T cell exhaustion in type 1 diabetes. JCI Insight.

[85] Sinclair LV (2025). Autophagy repression by antigen and cytokines shapes mitochondrial, migration and effector machinery in CD8 T cells. Nat Immunol.

[86] Waibel M (2023). Baricitinib and β-cell function in patients with new-onset type 1 diabetes. N Engl J Med.

[87] Medina-Serpas MA (2026). Spatial transcriptomics from pancreas and local draining lymph node tissue reveals a lymphotoxin-β signature in human type 1 diabetes. Cell Rep.

[88] Tickotsky N (2017). McPAS-TCR: a manually curated catalogue of pathology-associated T cell receptor sequences. Bioinformatics.

[89] Vita R (2025). The Immune Epitope Database(IEDB): 2024 update. Nucleic Acids Res.

[90] Gao S (2022). Single-cell RNA sequencing coupled to TCR profiling of large granular lymphocyte leukemia T cells. Nat Commun.

[91] Stadinski BD (2016). Hydrophobic CDR3 residues promote the development of self-reactive T cells. Nat Immunol.

[92] Kratchmarov R (2018). TCF1 expression marks self-renewing human CD8(+) T cells. Blood Adv.

[93] Kim YJ (2023). CD5 expression dynamically changes during the differentiation of human CD8(+) T cells predicting clinical response to immunotherapy. Immune Netw.

[94] Christen U (2023). Combination treatment of a novel CXCR3 antagonist ACT-777991 with an anti-CD3 antibody synergistically increases persistent remission in experimental models of type 1 diabetes. Clin Exp Immunol.

[95] Boof ML (2023). Pharmacokinetics, pharmacodynamics and safety of the novel C-X-C chemokine receptor 3 antagonist ACT-777991: Results from the first-in-human study in healthy adults. Br J Clin Pharmacol.

[96] Campbell-Thompson M (2012). Network for Pancreatic Organ Donors with Diabetes(nPOD): developing a tissue biobank for type 1 diabetes. Diabetes Metab Res Rev.

[97] Drotar DM (2024). Impaired islet function and normal exocrine enzyme secretion occur with low inter-regional variation in type 1 diabetes. Cell Rep.

[98] Subrahmanyam PB, Maecker HT (2017). CyTOF measurement of immunocompetence across major immune cell types. Curr Protoc Cytom.

[99] Crowell HL (2020). An R-based reproducible and user-friendly preprocessing pipeline for CyTOF data. F1000Res.

[100] Hahne F (2009). flowCore: a Bioconductor package for high throughput flow cytometry. BMC Bioinformatics.

[101] Phipson B (2022). propeller: testing for differences in cell type proportions in single cell data. Bioinformatics.

[102] McGinnis CS (2019). DoubletFinder: doublet detection in single-cell RNA sequencing data using artificial nearest neighbors. Cell Syst.

[103] Mule MP (2022). Normalizing and denoising protein expression data from droplet-based single cell profiling. Nat Commun.

[104] Hao Y (2021). Integrated analysis of multimodal single-cell data. Cell.

[105] Borcherding N (2020). scRepertoire: An R-based toolkit for single-cell immune receptor analysis. F1000Res.

[106] Finak G (2015). MAST: a flexible statistical framework for assessing transcriptional changes and characterizing heterogeneity in single-cell RNA sequencing data. Genome Biol.

[107] Yu G, He QY (2016). ReactomePA: an R/Bioconductor package for reactome pathway analysis and visualization. Mol Biosyst.

[108] Borcherding N (2021). Mapping the immune environment in clear cell renal carcinoma by single-cell genomics. Commun Biol.

[109] Bankhead P (2017). QuPath: Open source software for digital pathology image analysis. Sci Rep.

[111] Smith JA (2025). Protocol for processing and analyzing multiplexed images improves lymphatic cell identification and spatial architecture in human tissue. STAR Protoc.

[112] Perry DJ (2023). A genomic data archive from the Network for Pancreatic Organ donors with Diabetes. Sci Data.

