# Supplementary material for: Immune dysregulation and stem-like CD8+ T cell enrichment in type 1 diabetes pancreatic lymph nodes
Source: J Clin Invest. 2026 Sep 15;136(18):e196445. doi: 10.1172/JCI196445 (PMC13574167; doi:10.1172/JCI196445)
Supplement: Supplementary file 1 [file jci-136-196445-s232.pdf]

Figure S1

A

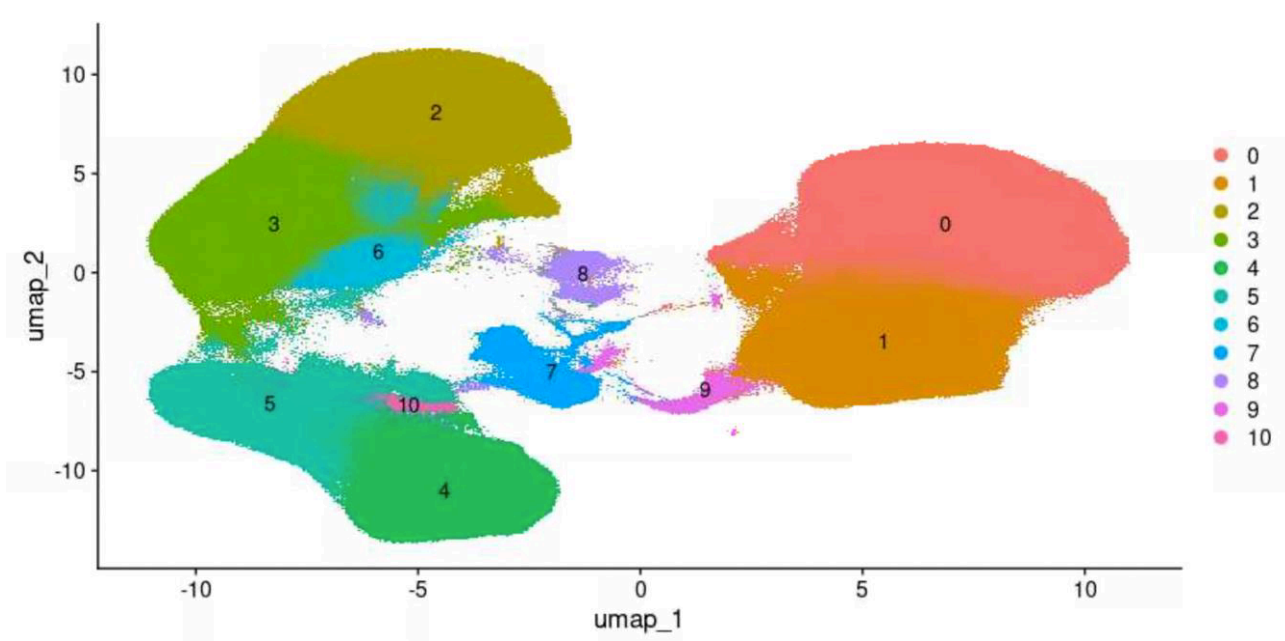
umap_2
umap_1
10
5
0
-5
-10
-10
-5
0
5
10
0
1
2
3
4
5
6
7
8
9
10

B

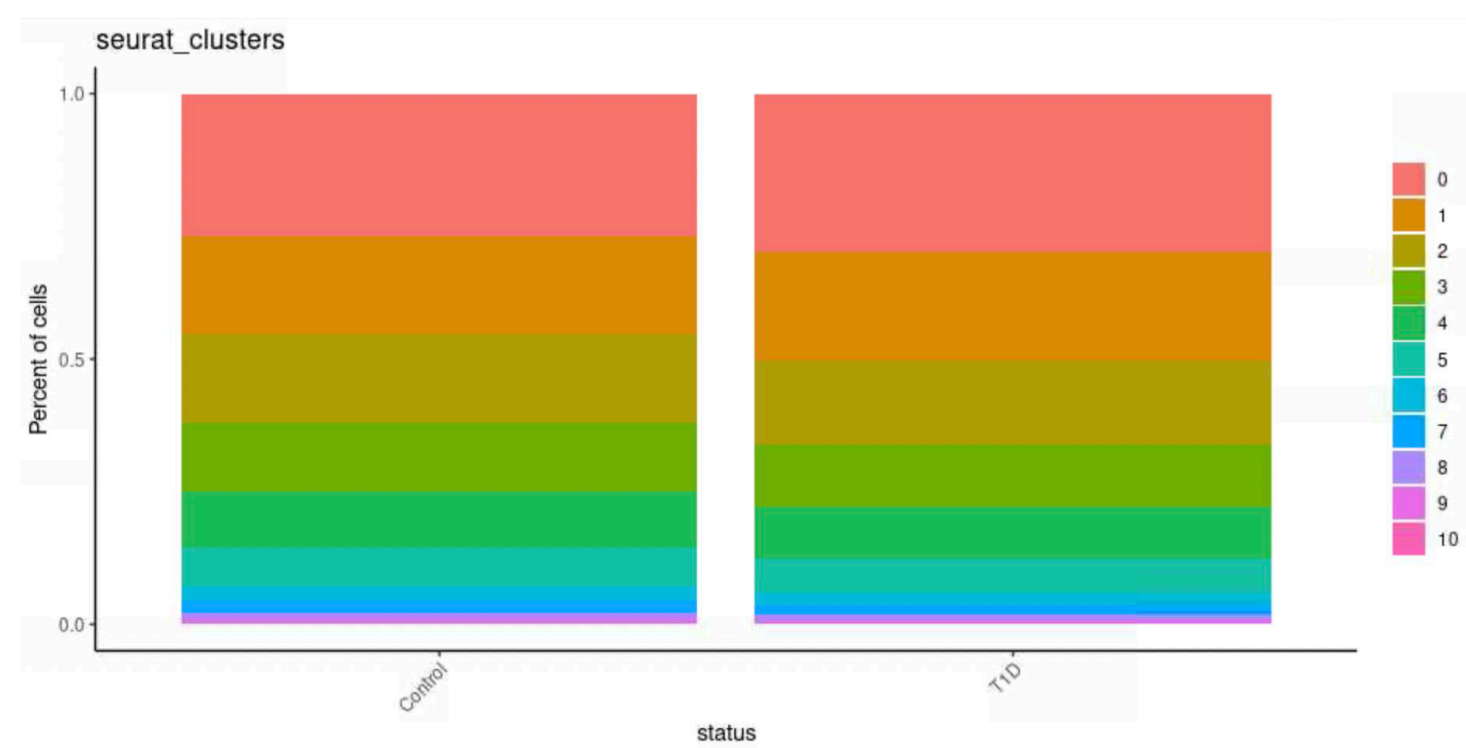
seurat_clusters
Percent of cells
1.0
0.5
0.0
Control
T1D
status
0
1
2
3
4
5
6
7
8
9
10

Figure S2

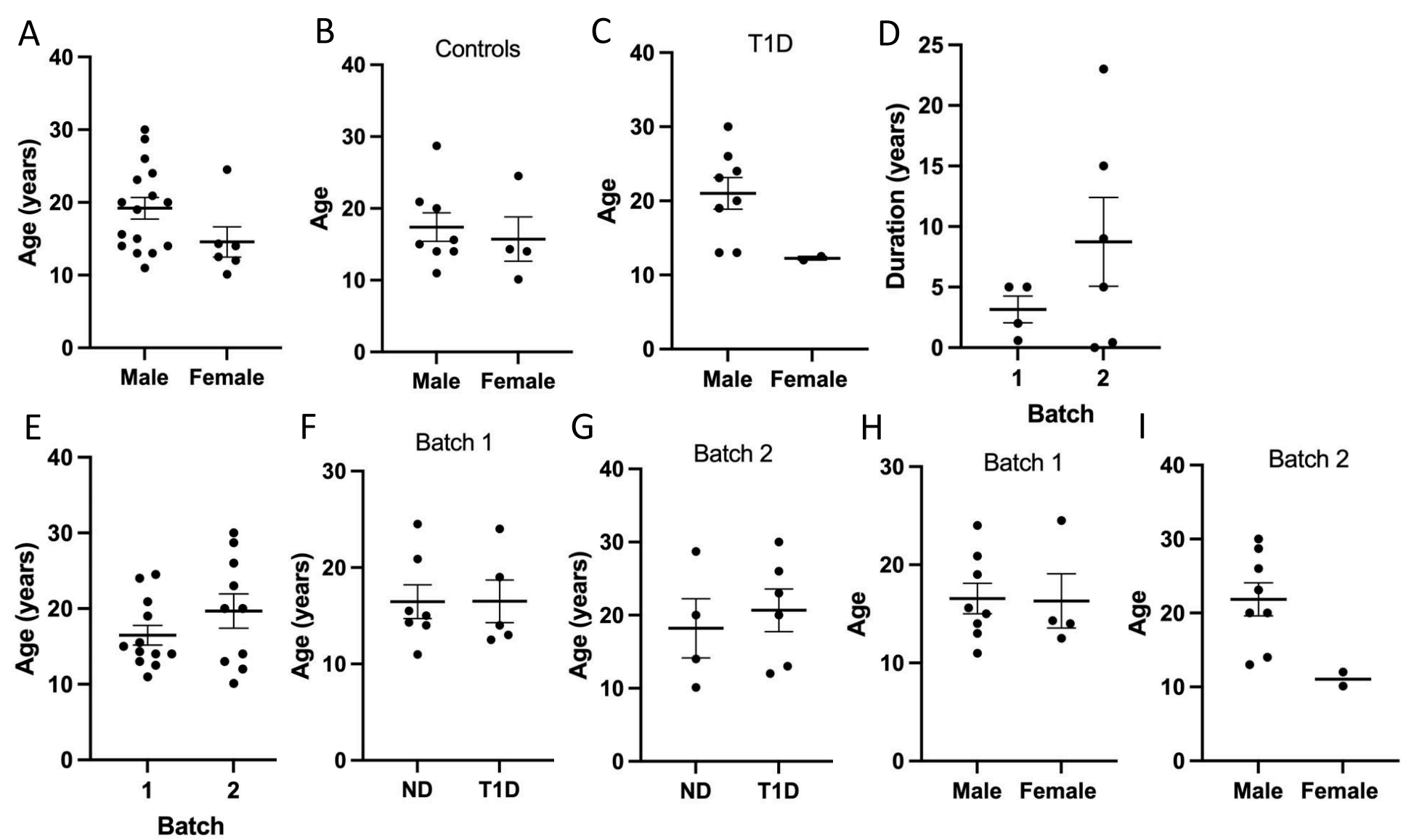

A
B
Controls
C
T1D
D
Age (years)
Age
Duration (years)
Male
Female
Batch
E
F
Batch 1
G
Batch 2
H
Batch 1
I
Batch 2
ND
T1D

Figure S3

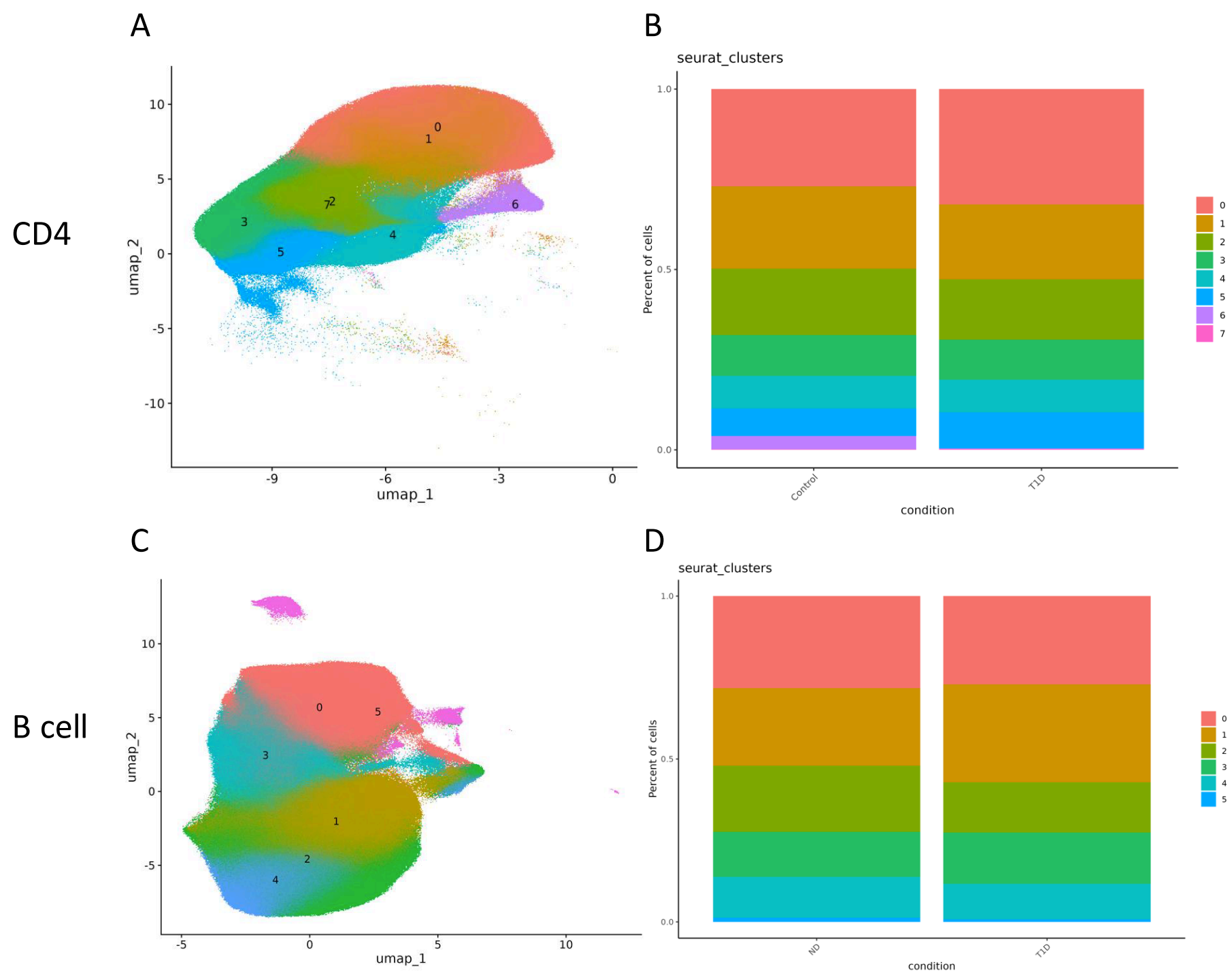
A
B
C
D
CD4
B cell
umap_1
umap_2
seurat_clusters
Percent of cells
condition
Control
T1D
ND

Figure S4

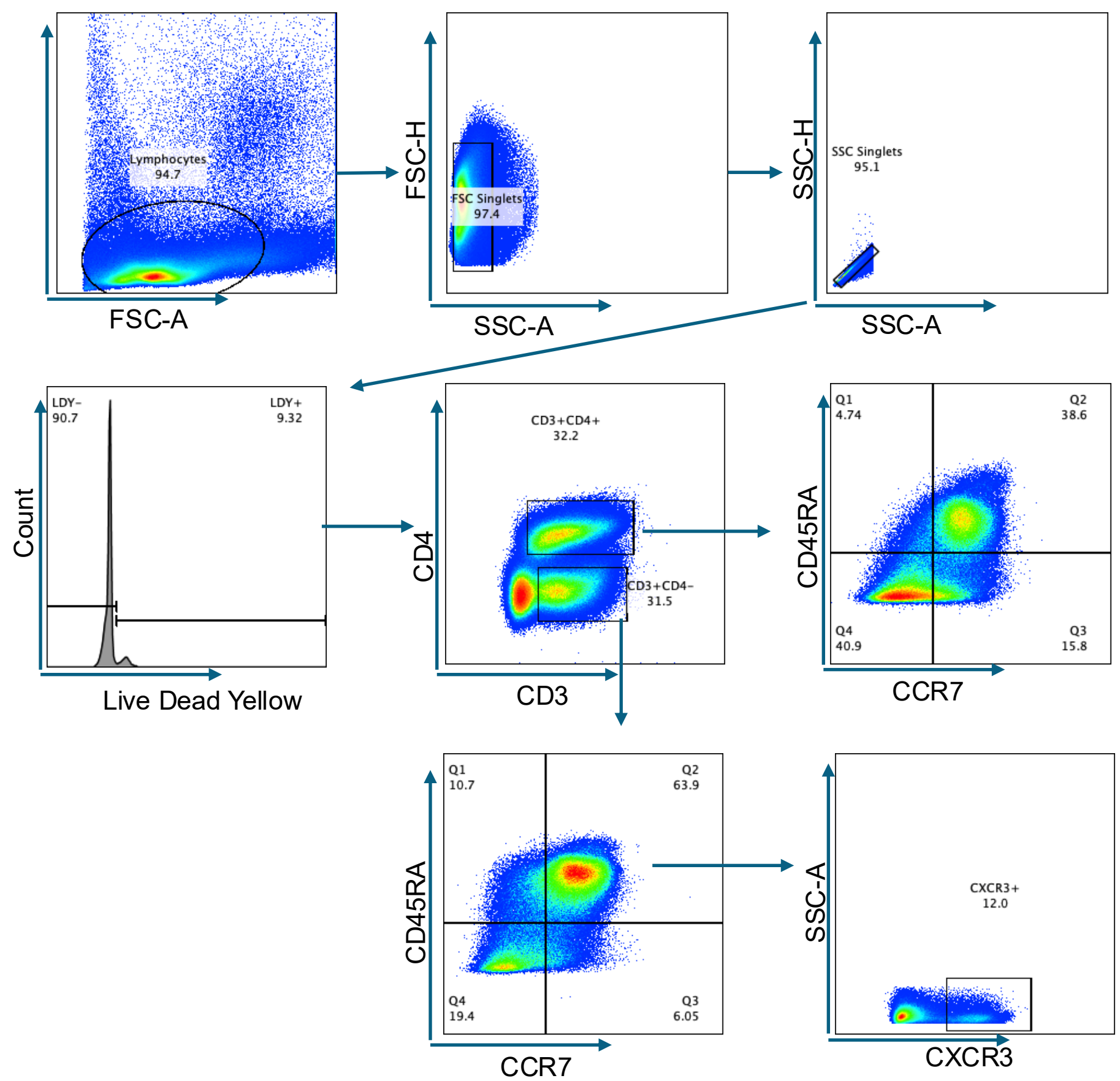

Lymphocytes
94.7
FSC-A
FSC-H
FSC Singlets
97.4
SSC-A
SSC-H
SSC Singlets
95.1
SSC-A
Count
LDY-
90.7
LDY+
9.32
Live Dead Yellow
CD4
CD3+CD4+
32.2
CD3+CD4-
31.5
CD3
CD45RA
Q1
4.74
Q2
38.6
Q4
40.9
Q3
15.8
CCR7
CD45RA
Q1
10.7
Q2
63.9
Q4
19.4
Q3
6.05
CCR7
SSC-A
CXCR3+
12.0
CXCR3

Figure S5

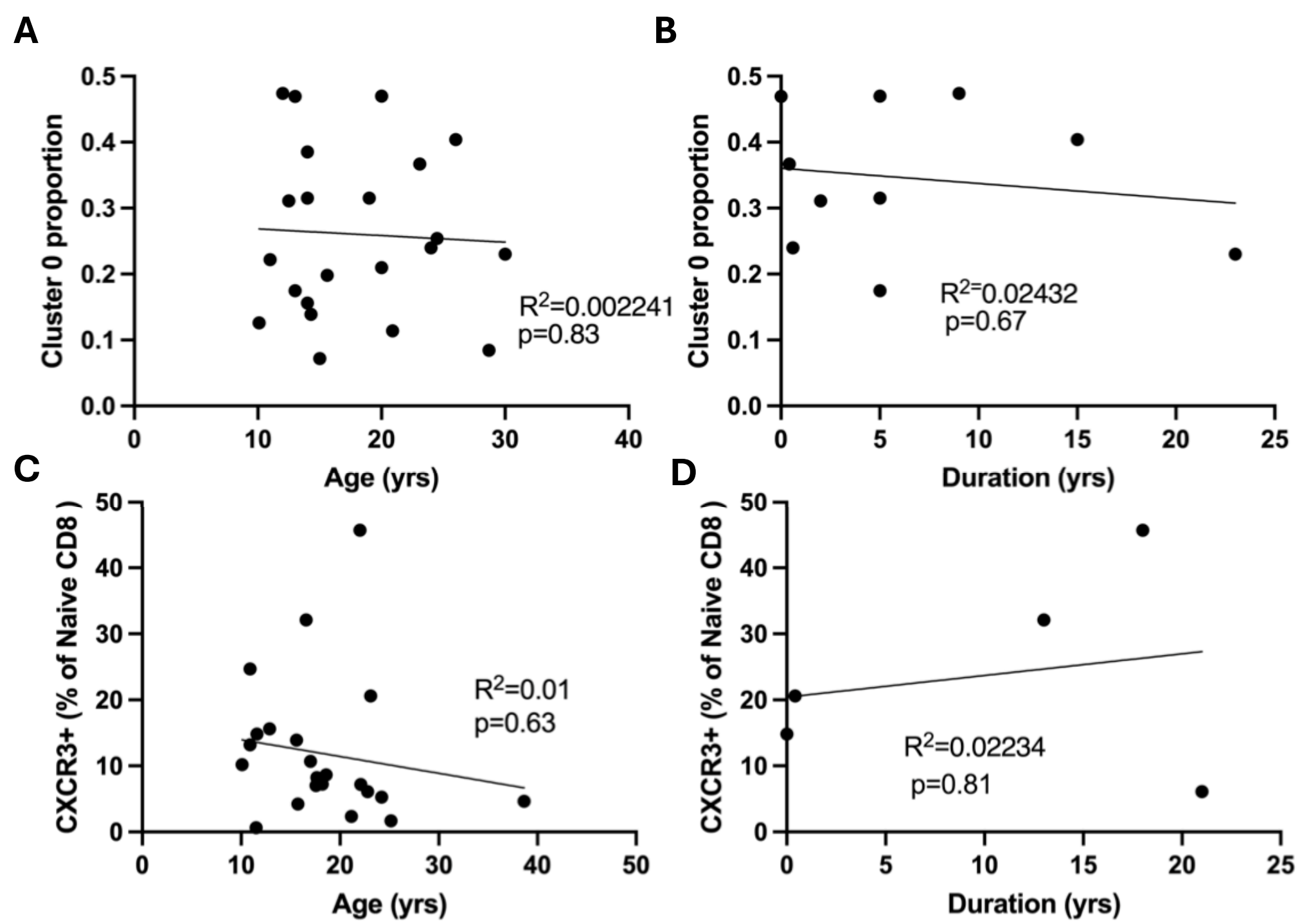
A
Cluster 0 proportion
Age (yrs)
R2=0.002241
p=0.83
B
Cluster 0 proportion
Duration (yrs)
R2=0.02432
p=0.67
C
CXCR3+ (% of Naive CD8)
Age (yrs)
R2=0.01
p=0.63
D
CXCR3+ (% of Naive CD8)
Duration (yrs)
R2=0.02234
p=0.81

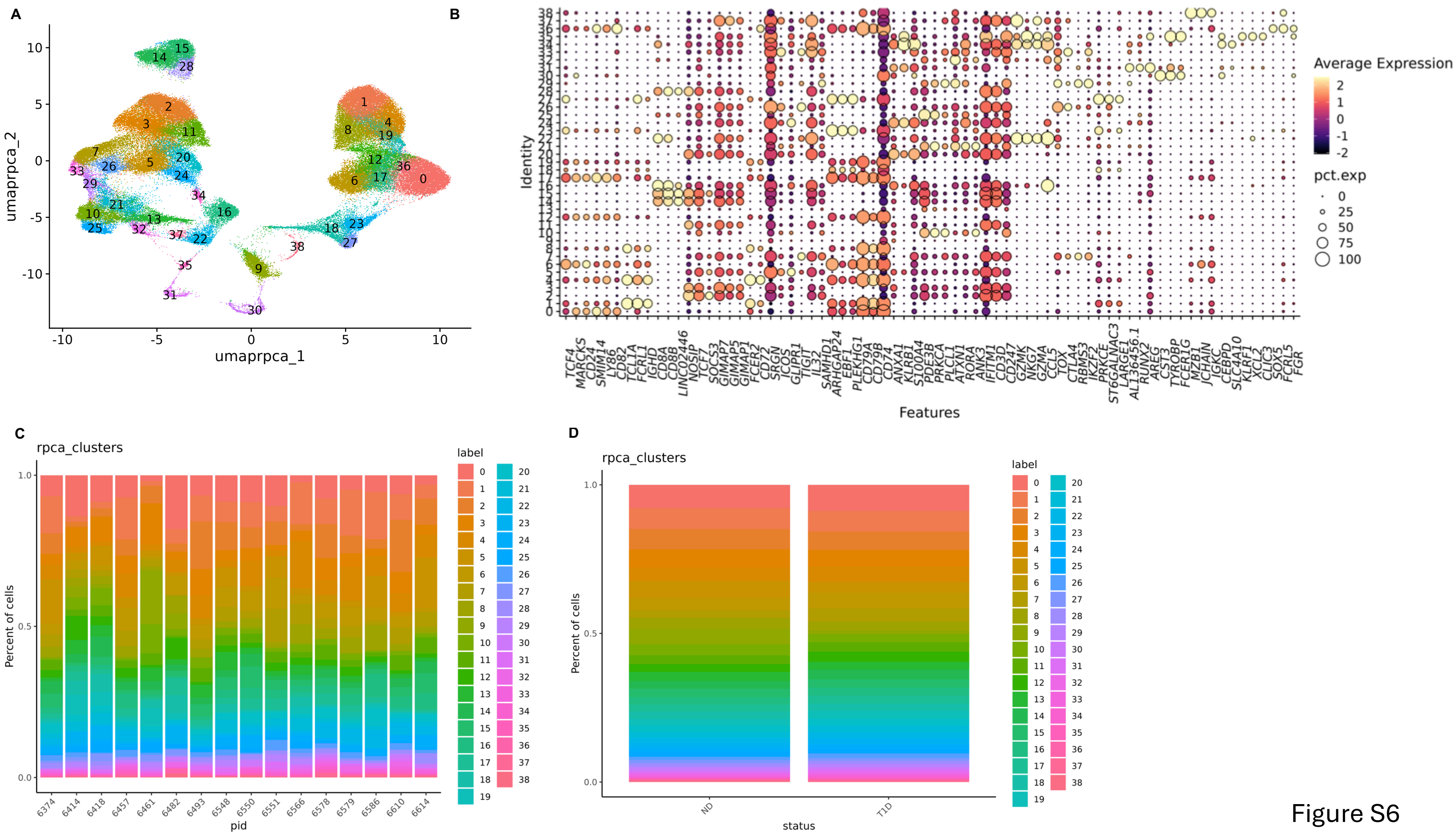
A
umaprpca_1
umaprpca_2
B
Average Expression
pct.exp
Identity
Features
TCF4 MARCKS CD24 SMIM14 LY86 CD82 TCL1A FCRL1 IGHD CD8B CD8A LINC02446 NOSIP TCF7 SOCS3 GIMAP7 GIMAP5 GIMAP1 FCER2 CD27 SRGN ICOS GLIPR1 TIGIT IL32 SAMHD1 ARHGAP24 EBF1 PLEKHG1 CD79A CD79B CD74 ANXA1 KLRB1 S100A4 PDE3B PRKCA PLCL1 ATXN1 RORA ANK3 IFITM1 CD3E CD247 GZMK NKG7 GZMA CCL5 TOX CTLA4 RBMS3 IKZF2 PRKCE ST6GALNAC3 LARGE1 AL136456.1 RUNX2 AREG CST3 TYROBP FCER1G MZB1 JCHAIN IGKC CEBPD SLC4A10 KLRF1 XCL2 CLIC3 SOX5 FCRL5 FGR
C
rpca_clusters
Percent of cells
pid
6374 6414 6418 6457 6461 6482 6493 6548 6550 6551 6566 6578 6579 6586 6610 6614
label
D
rpca_clusters
Percent of cells
status
ND
T1D
label

Figure S6

A

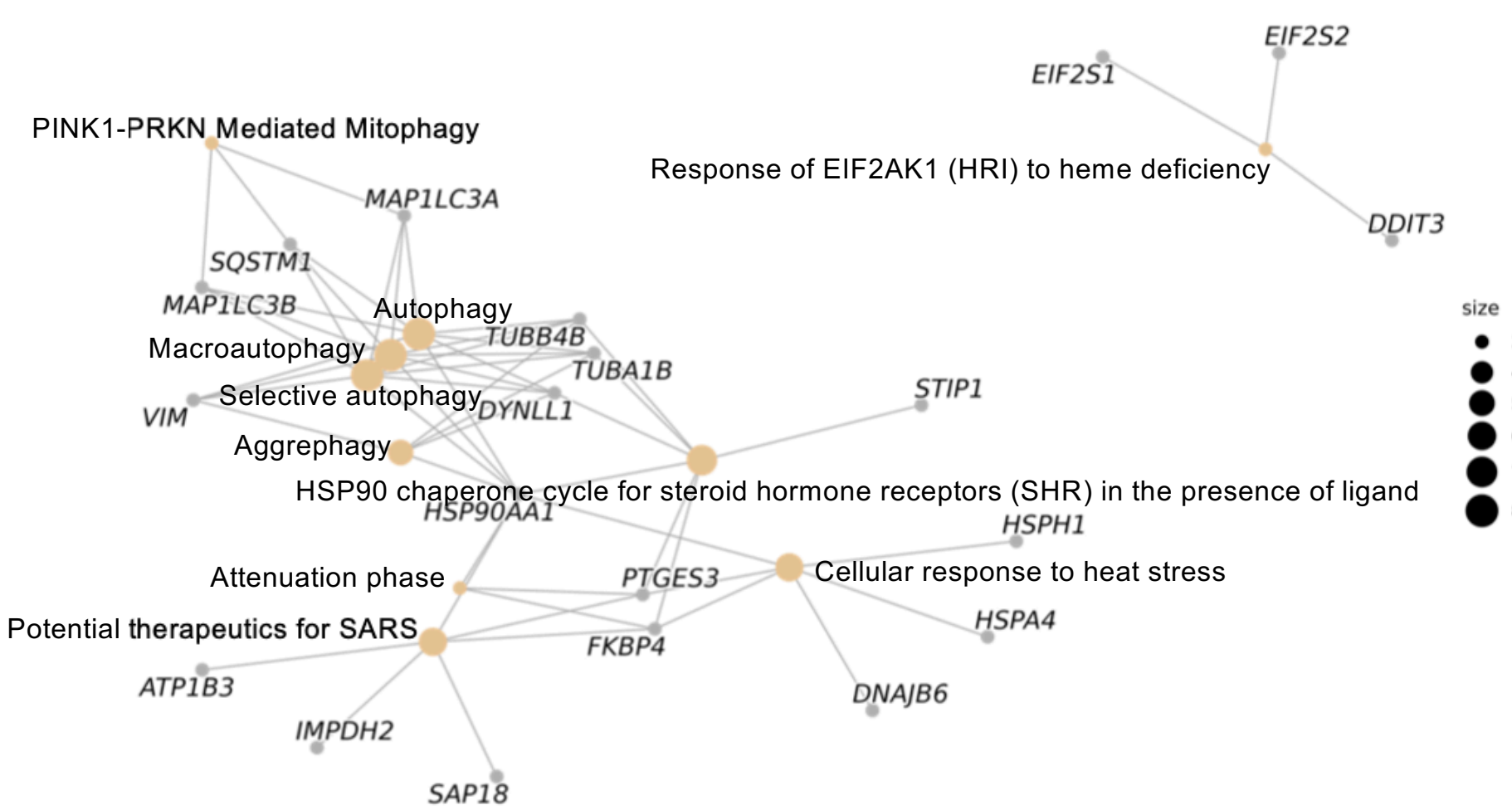
PINK1-PRKN Mediated Mitophagy
MAP1LC3A
SQSTM1
MAP1LC3B
Autophagy
TUBB4B
Macroautophagy
TUBA1B
Selective autophagy
DYNLL1
VIM
Aggrephagy
STIP1
HSP90 chaperone cycle for steroid hormone receptors (SHR) in the presence of ligand
HSP90AA1
HSPH1
Attenuation phase
PTGES3
Cellular response to heat stress
Potential therapeutics for SARS
FKBP4
HSPA4
ATP1B3
IMPDH2
DNAJB6
SAP18
EIF2S1
EIF2S2
Response of EIF2AK1 (HRI) to heme deficiency
DDIT3
size
3
4
5
6
7
8

B

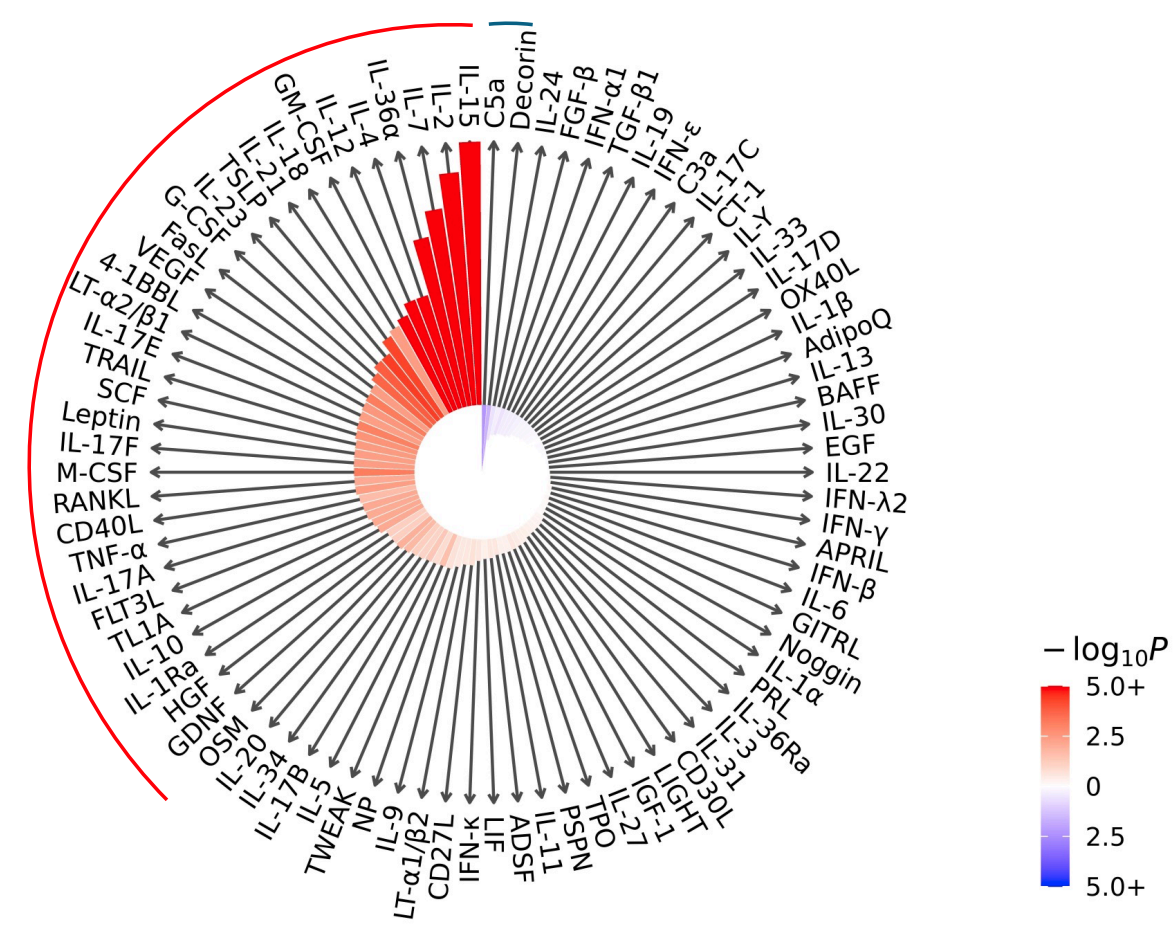
− log10P
5.0+
2.5
0
2.5
5.0+

Figure S7

Figure S8

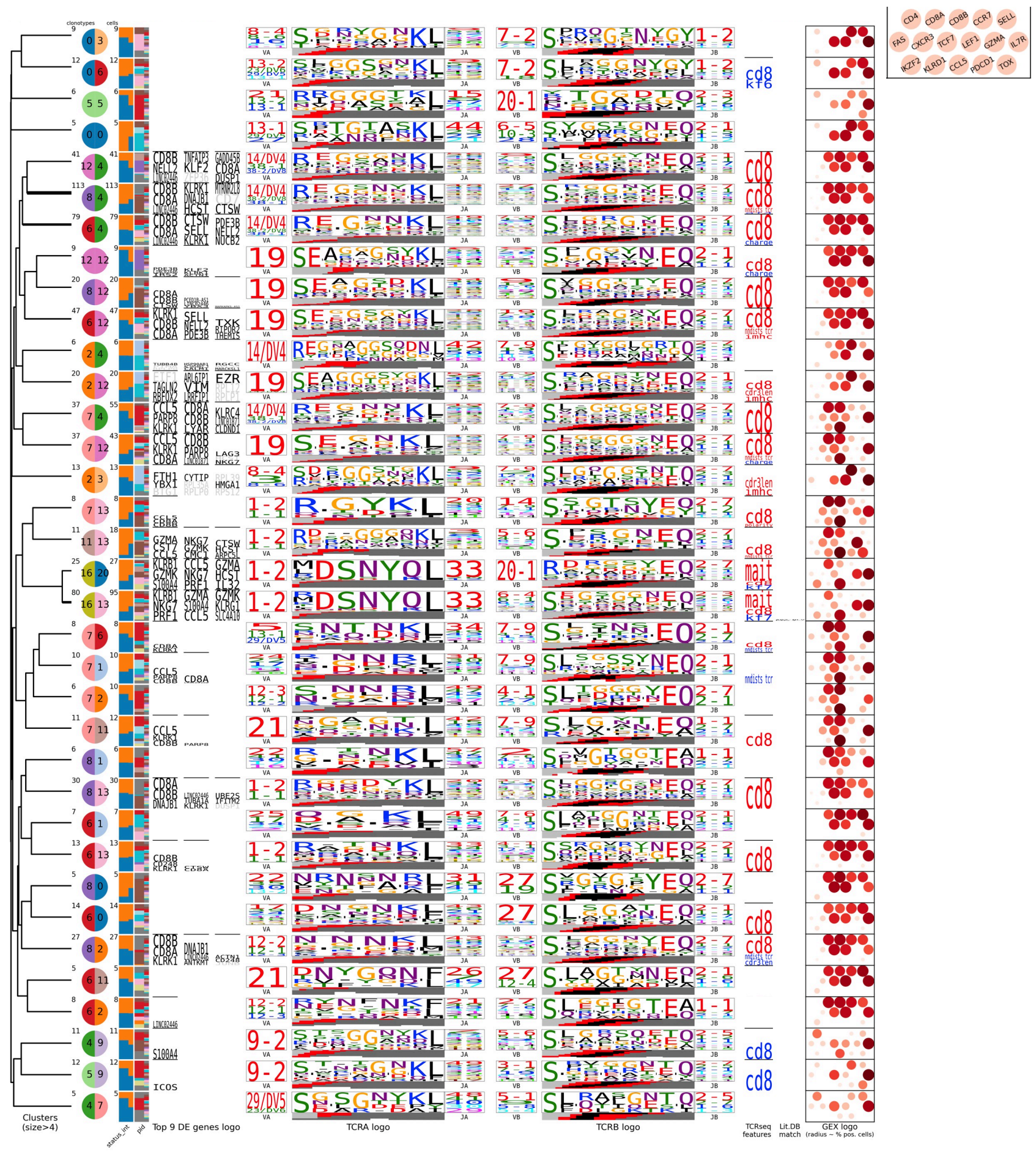
Clusters (size>4)
Top 9 DE genes logo
TCRA logo
TCRB logo
TCRseq features
Lit.DB match
GEX logo (radius ~ % pos. cells)
CD4
CD8A
CD8B
CCR7
SELL
FAS
CXCR3
TCF7
LEF1
GZMA
IL7R
IKZF2
KLRD1
CCL5
PDCD1
TOX

Figure S9

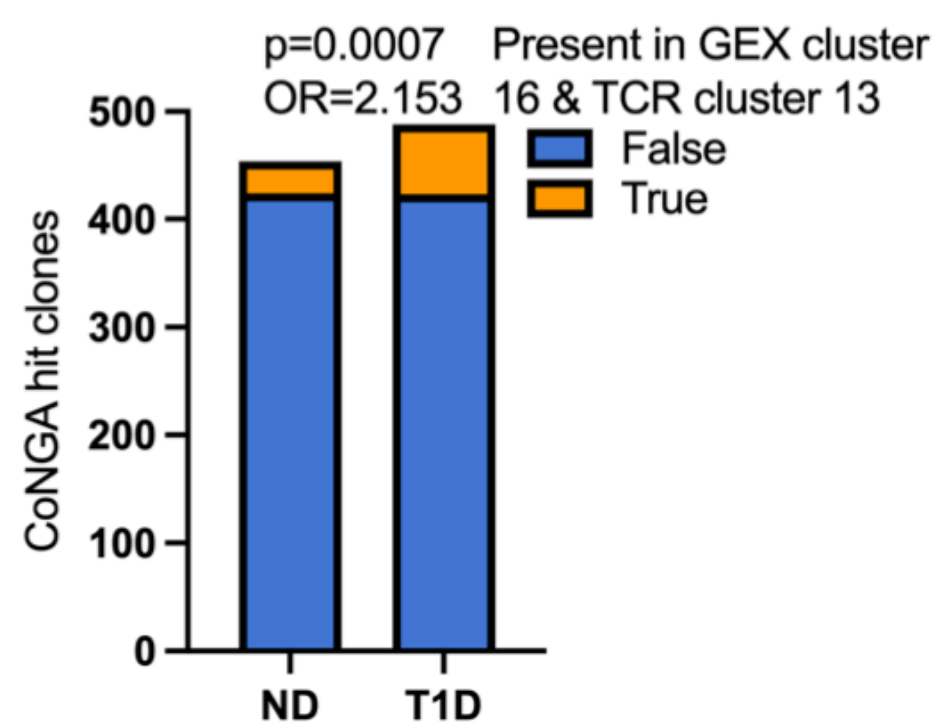

p=0.0007
OR=2.153
Present in GEX cluster 16 & TCR cluster 13
False
True
CoNGA hit clones
500
400
300
200
100
0
ND
T1D

Figure S10

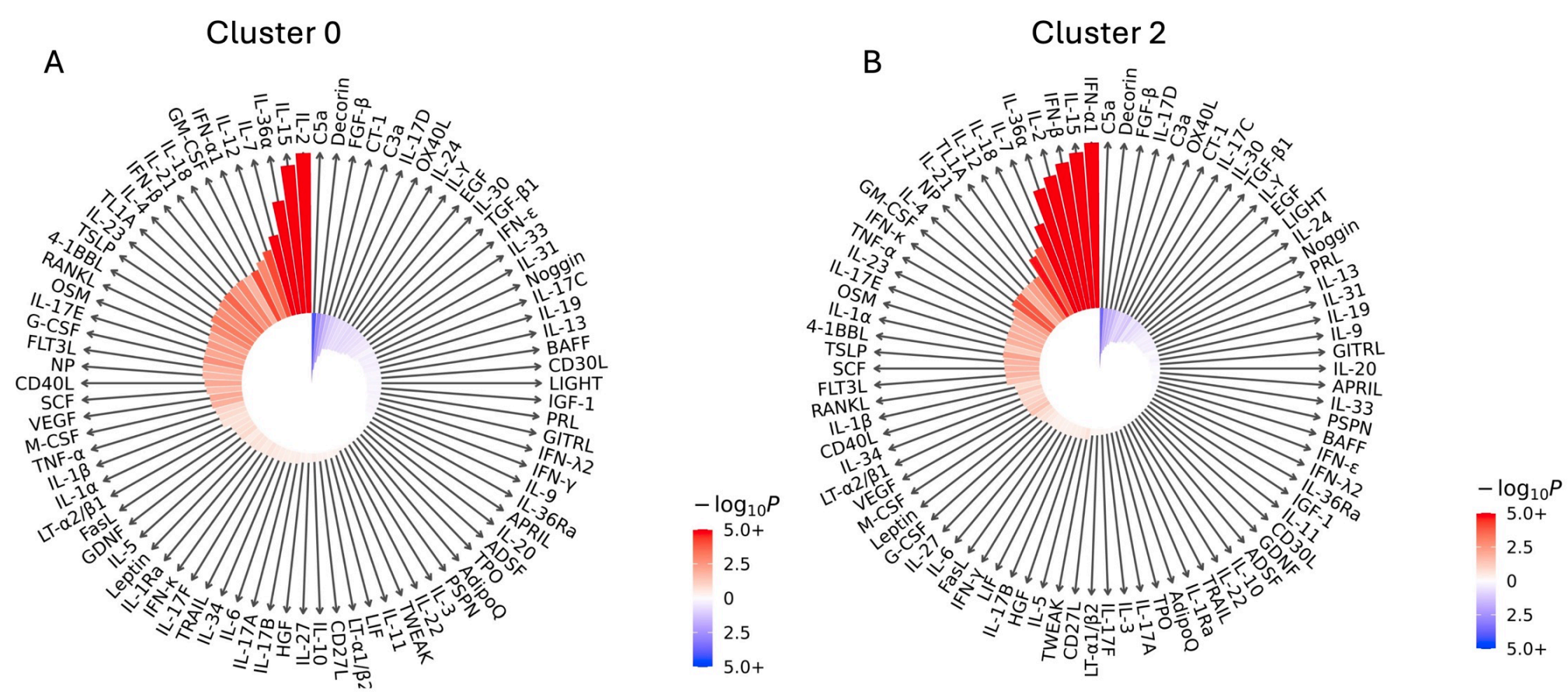

Figure S11

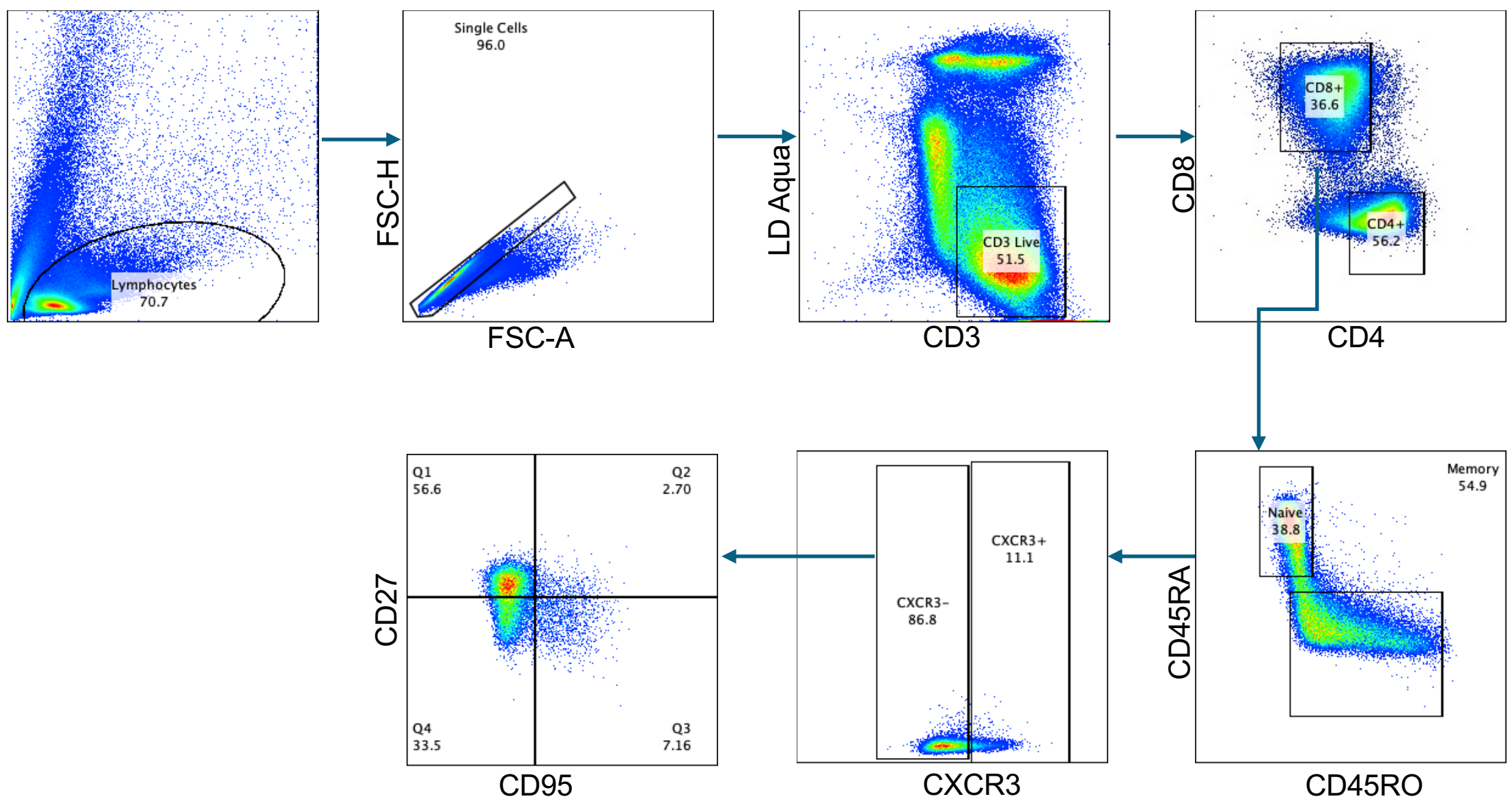

Lymphocytes
70.7
Single Cells
96.0
FSC-H
FSC-A
CD3 Live
51.5
LD Aqua
CD3
CD8+
36.6
CD4+
56.2
CD8
CD4
Memory
54.9
Naive
38.8
CD45RA
CD45RO
CXCR3-
86.8
CXCR3+
11.1
CXCR3
Q1
56.6
Q2
2.70
Q4
33.5
Q3
7.16
CD27
CD95

Figure S12

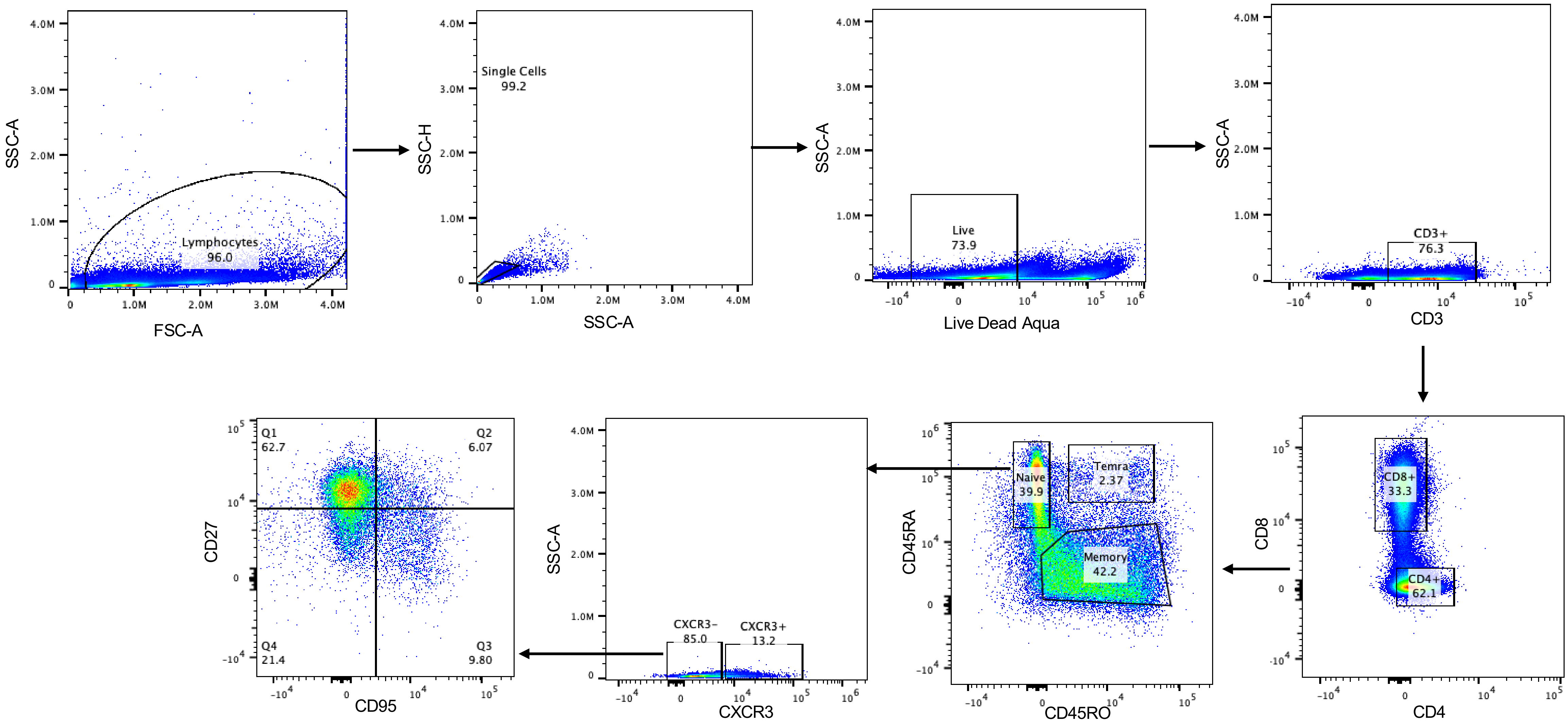

Lymphocytes
96.0
FSC-A
SSC-A
Single Cells
99.2
SSC-A
SSC-H
Live
73.9
Live Dead Aqua
SSC-A
CD3+
76.3
CD3
SSC-A
CD8+
33.3
CD4+
62.1
CD4
CD8
Naive
39.9
Temra
2.37
Memory
42.2
CD45RO
CD45RA
CXCR3-
85.0
CXCR3+
13.2
CXCR3
SSC-A
Q1
62.7
Q2
6.07
Q3
9.80
Q4
21.4
CD95
CD27

Figure S13

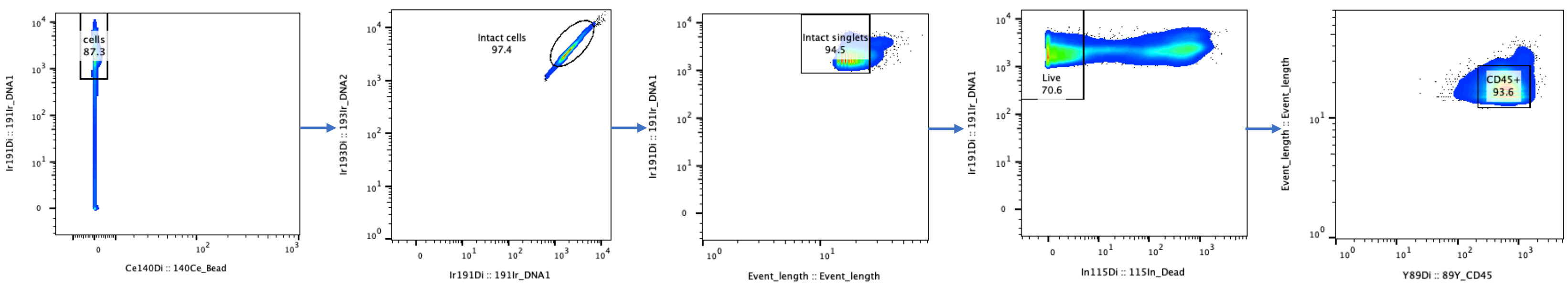

cells
87.3
Ir191Di :: 191Ir_DNA1
Ce140Di :: 140Ce_Bead
Intact cells
97.4
Ir193Di :: 193Ir_DNA2
Ir191Di :: 191Ir_DNA1
Intact singlets
94.5
Ir191Di :: 191Ir_DNA1
Event_length :: Event_length
Live
70.6
Ir191Di :: 191Ir_DNA1
In115Di :: 115In_Dead
CD45+
93.6
Event_length :: Event_length
Y89Di :: 89Y_CD45

Figure S14

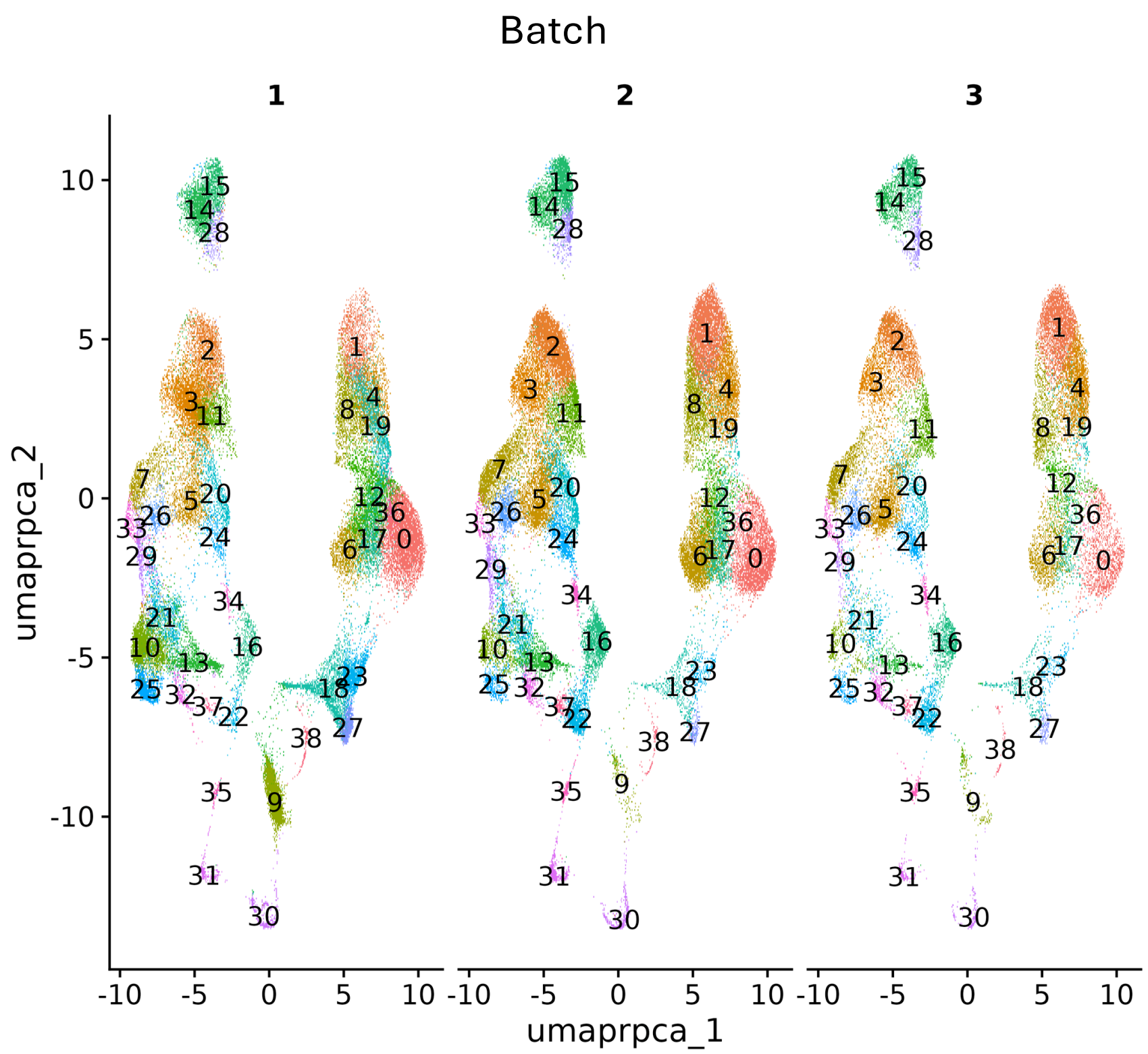

Figure S1. Similar profiles of total immune cells in the pLN between ND and T1D donors by CyTOF. A) UMAP illustrating clustering of total CD45+ pLN cells. B) Barplot of CyTOF clusters split by clinical status shows similar composition of immune cell subsets.

Figure S2. Covariate information in cyTOF batches. A) Age of the entire cohort separated by biological sex. B) Age of only controls in the cohort separated by biological sex. C) Age of only T1D donors in the cohort separated by biological sex. D) T1D duration separated by batch. E) Age of the entire cohort separated by batch. F) Age of donors in batch 1 separated by clinical status. G) Age of donors in batch 2 separated by clinical status. H) Age of donors in batch 1 separated by biological sex. I) Age of donors in batch 2 separated by biological sex. All nonsignificant results after performing either two-tailed T test or Mann Whitney test.

Figure S3. Similar frequency of CD4 and B cell cyTOF clusters by clinical status. A) UMAP of CD4 T cell cyTOF data. B) Barplot of clusters from A separated by clinical status. C) UMAP of B cell cyTOF data. D) Barplot of clusters from C separated by clinical status

Figure S4. Gating scheme for assessing CXCR3+ T cells in pLN by flow cytometry. Live, single cells were gated prior to excluding dead cells, followed by discrimination of CD4 and CD8 T cells. CD3+ CD4- cells were gated for naïve markers CD45RA and CCR7, and CXCR3+ cells were gated.

Figure S5. No significant association of naïve CXCR3$^+$ subsets with disease duration or age. A) Correlation of CyTOF cluster 0 proportion with age in all donors. B) Correlation of CyTOF cluster 0 proportion with disease duration in T1D donors only. C) Correlation of flow cytometry CXCR3$^+$ naïve CD8 T cell population with age in all donors. D) Correlation of flow cytometry CXCR3$^+$ naïve CD8 T cell population with disease duration in only T1D donors. P and R^2 values are the result of Pearson correlation.

Figure S6. No significant changes in scRNAseq clusters by clinical status. A) UMAP of pLN scRNAseq data. B) Dotplot showing average expression and percent expression of marker genes per cluster. C) Barplot of cluster proportion per donor. D) Barplot of cluster proportion by clinical status.

Figure S7. Naïve T cell pathway and immune response enrichment analysis (IREA) analysis. A) Reactome analysis of naïve CD8 T cells indicates upregulation of autophagy and mitophagy in T1D pLN. B) IREA analysis of naïve CD8 T cells implicates IL-15 as a driver of altered phenotypes. Differentially expressed genes of $p<0.05$ and Log2FC>0.26 were used as input into these analyses. Pathway analysis was performed with the ReactomePA R package, and results are reported when $p<0.05$. Bar height in the compass plot of IREA results is indicative of enrichment score, while color corresponds to FDR-adjusted P value (two-sided Wilcoxon rank-sum test). Red bar indicates which pathways were significantly upregulated, while the blue bar indicates the pathways that were significantly downregulated in this cluster.

Figure S8. CoNGA hits with clone size >4 in pLN. Semicircles indicate gene expression and TCR cluster, from left to right. Bar graphs illustrate the proportion of the cluster per status (leftmost bar) and dataset per status (right most bar). Orange=T1D, Blue=ND. Differentially expressed genes from each cluster are shown in the logo plot. TCRa and b gene information, CDR3a and CDR3b logos are provided per cluster. TCR features are also displayed.

Figure S9. Enriched cluster of MAIT T cells in T1D pLN. Barplot showing number of conga hit clones falling into GEX cluster 15 and TCR cluster 14, a MAIT T cell cluster, by clinical status. P value is the result of a two-way fishers exact test.

Figure S10. IREA Analysis of pLN vs pancreas CD8 T cells. A) Compass plot of IREA results using the top genes upregulated in pancreas in $CD8^+$ cluster 0 cells ($p<0.05$, $log2FC>0.26$) as input. B) Compass plot of IREA results using the top genes upregulated in pancreas in $CD8^+$ cluster 2 cells ($p<0.05$, $log2FC>0.26$) as input. Bar height is indicative of enrichment score, while color corresponds to FDR-adjusted P value (two-sided Wilcoxon rank-sum test).

Figure S11. Gating scheme for panel assessing stemness features. Flow cytometry of cryopreserved pLN cells was performed to assess expression of canonical Tscm markers Single lymphocytes were gated prior to live CD3+ cells, and CD4 and CD8 T cell discrimination. Memory and naïve T cells were gated, followed by discrimination of CXCR3- and CXCR3+ cells, and bonafide TSCM cells (CD27+CD95+) were gated from the CXCR3- fraction.

Figure S12. Gating scheme for panel assessing effector cytokine production by subsets in the pLN. Flow cytometry of cryopreserved pLN was performed after activation for 4 hours in the presence of PMA/ionomycin and golgistop to assess expression of cytokines. The same populations as above were gated to assess cytokine production.

Figure S13. Gating scheme for CyTOF data. Cells were gated from any residual beads after bead removal with premessa, followed by intact cells, intact singlets, live cells, and CD45+cells.

Figure S14. No significant differences in pLN cluster frequency across technical batches. UMAP of pLN scRNAseq data split by batches.

Table S1. Demographic information for mass cytometry (CyTOF) cohort.

Clinical Status	n	% Male	Age (yr)	% AAB+	Disease Duration (yr)	% Insulitis +	% High Risk HLA
T1D	10	80	19.5(18)	90	5(23)	90	100
ND	12	66.7	14.65(18.6)	0	NA	0	66.7

Age and disease duration represented as median(range)
*High Risk HLA was defined by possession of risk associated alleles:
DRB1*0401,0402,0403,0404;DRB1*0301

Table S2. Demographic information for flow cytometry validation cohort.

Clinical Status	n	% Male	Age (yr)	# AAB	Disease Duration (yr)	% Insulitis +	% High Risk HLA
T1D	5	60	19.2±5.0	20% negative, 20% single, 20% double, 40% triple	10.5±9.8	80	100
AAB+	4	75	21.5±3.1	50% single, 50% double	NA	25	75
ND	14	64	17.1±7.3		NA	0	60

*High Risk HLA was defined by possession of risk associated alleles:
DRB1*0401,0402,0403,0404,0407;DRB1*0301

Table S3. Demographic information for scRNAseq cohort.

Clinical Status	n	% Male	Age (yr)	% AAB+	Disease Duration (yr)	% Insulitis+	% High Risk HLA
T1D	9	55	17.0±6.5	100	1.80±3.4	100	100
ND	7	71.4	17.1±3.1	0	NA	0	80

*High Risk HLA was defined by possession of risk associated alleles:
DRB1*0401,0402,0403,0404;DRB1*0301

Table S4. Differentially expressed genes between T1D (positive fold change) and ND (negative fold change) in T cell clusters (see supplemental values).

Table S5. CoNGA identified clones from clusters found to be enriched in T1D (see supplemental values).

Table S6. nPOD donor and corresponding experiment information.

case_id	RR_id	donor_type	experiment
6374	SAMN15879427	No Diabetes	scRNAseq, CyTOF
6131	SAMN15879188	No Diabetes	Fresh flow cytometry
6174	SAMN15879230	No Diabetes	CyTOF, flow confirmation
6178	SAMN15879234	No Diabetes	CyTOF
6212	SAMN15879268	Type 1 Diabetes	CyTOF, flow confirmation
6228	SAMN15879284	Type 1 Diabetes	CyTOF

6238	SAMN15879294	No Diabetes	CyTOF
6243	SAMN15879299	Type 1 Diabetes	CyTOF
6247	SAMN15879303	Type 1 Diabetes	CyTOF
6264	SAMN15879318	Type 1 Diabetes	CyTOF, flow confirmation
6266	SAMN15879320	Type 1 Diabetes	CyTOF
6282	SAMN15879336	No Diabetes	CyTOF
6306	SAMN15879360	Type 1 Diabetes	CyTOF
6317	SAMN15879371	No Diabetes	CyTOF
6330	SAMN15879384	Type 1 Diabetes	Fresh flow cytometry
6336	SAMN15879390	No Diabetes	CyTOF, flow confirmation
6341	SAMN15879395	Type 1 Diabetes	CyTOF
6371	SAMN15879424	Type 1 Diabetes	CyTOF, flow confirmation
6375	SAMN15879428	No Diabetes	CyTOF
6380	SAMN15879433	Type 1 Diabetes	Fresh flow cytometry
6384	SAMN15879437	No Diabetes	Fresh flow cytometry
6385	SAMN15879438	No Diabetes	Fresh flow cytometry
6386	SAMN15879439	No Diabetes	CyTOF
6387	SAMN15879440	No Diabetes	CyTOF, fresh flow cytometry, flow confirmation
6389	SAMN15879442	No Diabetes	Fresh flow cytometry
6391	SAMN15879444	No Diabetes	CyTOF
6393	SAMN15879446	No Diabetes	Fresh flow cytometry
6397	SAMN15879450	Autoantibody Positive	Fresh flow cytometry
6400	SAMN15879453	Autoantibody Positive	Fresh flow cytometry
6410	SAMN15879463	Other - No Diabetes	Fresh flow cytometry
6412	SAMN15879465	No Diabetes	Fresh flow cytometry
6413	SAMN15879466	No Diabetes	CyTOF,Fresh flow cytometry, flow confirmation
6414	SAMN15879467	Type 1 Diabetes	scRNAseq, CyTOF, Fresh flow cytometry
6415	SAMN15879468	No Diabetes	Fresh flow cytometry
6417	SAMN15879470	No Diabetes	Fresh flow cytometry
6418	SAMN15879471	Type 1 Diabetes	scRNAseq
6420	SAMN15879473	No Diabetes	Fresh flow cytometry
6422	SAMN15879475	Type 1 Diabetes	Fresh flow cytometry
6424	SAMN15879477	Autoantibody Positive	Fresh flow cytometry
6425	SAMN15879478	No Diabetes	Fresh flow cytometry
6429	SAMN15879482	Autoantibody Positive	Fresh flow cytometry
6432	SAMN15879485	Type 1 Diabetes	Fresh flow cytometry
6440	SAMN15879493	No Diabetes	Fresh flow cytometry
6457	SAMN15879510	Type 1 Diabetes	scRNAseq

6461	SAMN15879514	No Diabetes	scRNAseq
6462	SAMN15879515	No Diabetes	Flow confirmation
6482	SAMN15879535	No Diabetes	scRNAseq
6493	SAMN15879546	No Diabetes	scRNAseq
6536	SAMN18242780	Type 1 Diabetes	scRNAseq
6548	SAMN25652259	No Diabetes	scRNAseq
6550	SAMN25652261	Type 1 Diabetes	scRNAseq
6551	SAMN25652262	Type 1 Diabetes	scRNAseq
6566	SAMN33284286	Type 1 Diabetes	scRNAseq
6578	SAMN33284295	Type 1 Diabetes	scRNAseq, Flow confirmation
6579	SAMN33284296	Type 1 Diabetes	scRNAseq
6586	SAMN38117306	No Diabetes	scRNAseq
6606	SAMN40555593	Type 1 Diabetes	Flow confirmation
6610	SAMN44486496	Type 1 Diabetes	scRNAseq
6614	SAMN44486500	No Diabetes	scRNAseq

Table S7. Flow cytometry panel for broad T cell phenotype.

Antigen	Fluorophore	Clone	Manufacturer
CCR7	BV421	G043H7	Biolegend
CD4	FITC	RPA-T4	BioLegend
CD45RA	PerCP-Cy5.5	HI100	BioLegend
CXCR5	PE	J252D4	BioLegend
CXCR3	PE-Cy7	G025H7	BioLegend
PD-1	APC	eBioJ105	eBioscience
CD3	APC-H7	SK7	BD
Live/Dead	Live Dead Yellow	NA	Invitrogen

Table S8. Flow cytometry panel assessing stem-like and exhausted T cell features in pLN.

Marker	Fluorophore	Catalog	Vendor
Zombie Viability Die	Aqua	423101	Biolegend
CD45	Spark blue 574	368558	Biolegend
CD3	Spark YG593	344868	Biolegend
CD4	Spark UV387	344686	Biolegend
CD8	BUV805	568334	BD/Fisher

CD45RA	BUV563	612926	BD/Fisher
CD45RO	R718	567536	BD/Fisher
CD127	BV711	351328	Biolegend
PD1	BV605	329924	Biolegend
CXCR3	BV421	353716	Biolegend
CXCR5	AF647	356906	Biolegend
CD95	RB780	569126	BD/Fisher
CD27	BUV496	751678	BD/Fisher
CD57	BV785	393330	Biolegend
CD69	RB705	570278	BD/Fisher
CD28	BUV395	742530	BD/Fisher
CD5	RY703	771019	BD/Fisher
CD122	BB515	566059	BD
TCF1	PE	655207	Biolegend
TOX	AF647	568356	BD

Table S9. Flow cytometry panel examining pLN CD8^{+} T cell functionality

Marker	Fluorophore	Catalog	Vendor
Zombie Viability Die	Aqua	423101	Biolegend
CD3	Spark YG593	344868	Biolegend
CD4	Spark UV387	344686	Biolegend
CD8	APC	301014	Biolegend
CD45RA	BUV563	612926	BD/Fisher
CD45RO	R718	567536	BD/Fisher
CXCR3	BV421	353716	Biolegend
CD95	RB780	569126	BD/Fisher
CD27	BUV496	751678	BD/Fisher
CD28	BUV395	742530	BD/Fisher
IFNG	PeCy7	502528	Biolegend
TNFa	BV650	502938	Biolegend
IL2	BV750	500359	Biolegend

Table S10. Mass cytometry panel.

Antigen	Isotope	Clone	Source
CD45	Y89Di	HI30	Fluidigm
CD57	In113Di	HCD57	BioLegend
CD19	Nd142Di	SJ25C1	Southern Biotech
CD4	Nd143Di	SK3	BioLegend
CD8	Nd144Di	SK1	BioLegend
CD16	Nd145Di	3G8	Fluidigm
IgD	Nd146Di	IA6-2	BioLegend
CD20	Sm147Di	2H7	Fluidigm
CD14	Nd148Di	RMO52	Fluidigm
CD56	Sm149Di	NCAM16.2	Fluidigm
CD3	Nd150Di	UCHT1	BD
CD103	Eu151Di	Ber-ACT8	Fluidigm
TCRgd	Sm152Di	11F2	Fluidigm
CD45RA	Eu153Di	HI100	Fluidigm
TIGIT	Sm154Di	MBSA43	Fluidigm
CCR6	Gd155Di	11A9	BD
CXCR3	Gd156Di	G025H7	Fluidigm
CD86	Gd157Di	IT2.2	BioLegend
CD27	Gd158Di	L128	Fluidigm
CD11c	Tb159Di	Bu15	Fluidigm
CCR7	Gd160Di	150503	R&D Systems
CD69	Dy162Di	FN50	Fluidigm
CXCR5	Dy164Di	51505	Fluidigm
CD127	Ho165Di	A019D5	BioLegend
CD33	Er166Di	P67.8	BD
CD28	Er167Di	L293	BD
CD24	Er168Di	ML5	BioLegend
ICOS	Tm169Di	DX29	BD
CD161	Er170Di	DX12	BD
CD226	Yb171Di	DX11	Fluidigm
CD38	Yb172Di	HIT2	Fluidigm
CD123	Yb173Di	9F5	BD
PD1	Yb174Di	EH12.2H7	Fluidigm
HLA-DR	Lu175Di	G46-6	BD
CD25	Yb176Di	M-A251	BD
CD11b	Bi209Di	ICRF44	Fluidigm

Table S11. TotalSeq antibodies and dextramer information

Antibody/Dextramer	Item No	Dextramer Category
anti-human CD4	344651	
anti-human CD8	344753	
anti-human CD69	310951	
anti-human CD27	302853	

anti-human CD45RA	304163	
anti-human CD25	302649	
anti-human CD127 (IL-7Rα)	351356	
HLA-A 0201 NLVPMVATV		CMV
HLA-A 0201 VLEETSVML		CMV
HLA-A 0201 ILGFVFTLTV		MP
HLA-A 0201 KLGEFYNQMM		Influenza
DRB1 0401 PKYVKQNTLKLAT		Influenza
HLA-A 0101 VLFGLGFAI		IGRP
HLA-A 0101 HLVEALYLV		Insulin
HLA-A 0101 ALWGPDPAAA		Insulin
HLA-A 0101 VMNILLQYVV		GAD65
HLA-A 0101 MVWESGCTV		IA2
DRB1*0301 IAFTSEHSHFSLK		GAD65
DRB1*0301 NFIRMVISNPAAT		GAD65
DRB1*0301 GAGSLQPLALEGSLQKRG		Proinsulin
HLA-B*0801 AAKGRGAAL		General negative control
HLA-A*0201 ALIAPVHAV		
DRB1*0401 PVSKMRMATPLLMQA		CLIP peptide

Table S12. Phenocycler panel information.

	**Atto 550**			**Alexa Fluor 647**			**Alexa Fluor 750**		
**Cycle Order**	**Target**	**Barcode**	**Exposure Time (ms)**	**Target**	**Barcode**	**Exposure Time (ms)**	**Target**	**Barcode**	**Exposure Time (ms)**
1	IAPP	BX032	150	CD4	BX003	150	CD107a	BX006	150
2	CD31	BX001	125	CD68	BX015	100	SMA	BX013	100
3	CD44	BX005	150	CD99	BX010	75	Vimentin	BX022	100
4	ECAD	BX014	150	CD66	BX016	150	IDO1	BX027	150
5	INS	BX002	100	CD11c	BX024	60	Ker8-18	BX107	150
6	CD38	BX089	150	CD34	BX025	150	HLADR	BX033	150
7	iNOS	BX023	100	B3Tub	BX017	150	M2Gal3	BX035	80
8	CD8	BX026	150	FOXP3	BX031	150	PCNA	BX036	150
9	CD57	BX028	80	GranB	BX041	150	Ki67	BX047	150
10	HLAA	BX029	100	Col_IV	BX042	150	CD20	BX064	150
11	VISTA	BX040	150	PD-1	BX046	150	PanCK	BX066	75
12	LAG3	BX055	125	TCF-1	BX061	150	STT	BX037	150
13	TOX	BX060	150	ICOS	BX065	150	Caveolin	BX086	150

14	CD163	BX069	125	PD-L1	BX067	150	EpCAM	BX091	150
15	CD79a	BX090	125	CD3e	BX080	150	Ker5	BX101	125
16	GCG	BX020	75	Bcl-2	BX085	125	BetaActin	BX117	100
17	MPO	BX098	125	CD56	BX088	150	-		-
18	CD39	BX099	150	Iba1	BX021	100	-		-
19	SOX2	BX102	150	CD209	BX329	150	-		-
20	PDPN	BX121	150	CD11b	BX524	80	-		-
21	CD206	BX361	150	TP63	BX093	100	-		-
22	-	-	-	-	-	-	-		-
23	-	-	-	-	-	-	-		-
24	-	-	-	-	-	-	-		-
